# Playing Hide-and-Seek in Beta-Globin Genes: Gene Conversion Transferring a Beneficial Mutation between Differentially Expressed Gene Duplicates

**DOI:** 10.3390/genes9100492

**Published:** 2018-10-12

**Authors:** Michaela Strážnická, Silvia Marková, Jeremy B. Searle, Petr Kotlík

**Affiliations:** 1 Laboratory of Molecular Ecology, Institute of Animal Physiology and Genetics, Czech Academy of Sciences, Rumburská 89, 27721 Liběchov, Czech Republic; markova@iapg.cas.cz (S.M.); kotlik@iapg.cas.cz (P.K.); 2 Department of Zoology, Faculty of Science, Charles University, Viničná 7, 12844 Prague 2, Czech Republic; 3 Department of Animal Science and Food Processing, Faculty of Tropical AgriSciences, Czech University of Life Sciences Prague, Kamýcká 129, 165 00 Prague 6-Suchdol, Czech Republic; 4 Department of Ecology and Evolutionary Biology, Cornell University, Ithaca, NY 14853, USA; jeremy.searle@cornell.edu

**Keywords:** adaptive phylogeography, cysteine, antioxidative capacity, gene conversion, Chi motif, environmental selection

## Abstract

Increasing evidence suggests that adaptation to diverse environments often involves selection on existing variation rather than new mutations. A previous study identified a nonsynonymous single nucleotide polymorphism (SNP) in exon 2 of two paralogous β-globin genes of the bank vole (*Clethrionomys glareolus*) in Britain in which the ancestral serine (Ser) and the derived cysteine (Cys) allele represent geographically partitioned functional variation affecting the erythrocyte antioxidative capacity. Here we studied the geographical pattern of the two-locus Ser/Cys polymorphism throughout Europe and tested for the geographic correlation between environmental variables and allele frequency, expected if the polymorphism was under spatially heterogeneous environment-related selection. Although bank vole population history clearly is important in shaping the dispersal of the oxidative stress protective Cys allele, analyses correcting for population structure suggest the Europe-wide pattern is affected by geographical variation in environmental conditions. The β-globin phenotype is encoded by the major paralog HBB-T1 but we found evidence of bidirectional gene conversion of exon 2 with the low-expression paralog HBB-T2. Our data support the model where gene conversion reshuffling genotypes between high- and low- expressed paralogs enables tuning of erythrocyte thiol levels, which may help maintain intracellular redox balance under fluctuating environmental conditions. Therefore, our study suggests a possible role for gene conversion between differentially expressed gene duplicates as a mechanism of physiological adaptation of populations to new or changing environments.

## 1. Introduction

Recent studies increasingly suggest that adaptation to novel environments often involves selection on previously existing variation rather than new mutations [1,2,3,4,5]. Ecologically relevant variation at a genetic locus can be maintained within species if the same allele is adaptive in one environment and maladaptive in another [6]. Such adaptive polymorphisms may be viewed as reservoirs of functional variability within species [2,7] that could facilitate rapid evolutionary response to changing environmental conditions or during colonization of novel environments [8,9,10,11,12].

Amino acid variation at the genes coding for haemoglobin (Hb) subunits shows association with environment-related fitness difference in a diverse variety of organisms [13,14,15,16,17,18,19,20]. Recently, the function of Hb in evolutionary adaptation has been broadened beyond the increased oxygen affinity under hypoxia to include a role as a physiologically significant antioxidant, with the key role played by surface-exposed reactive cysteine (Cys) residues tuned to undergo oxidative modification [21,22,23,24]. These Cys residues are not directly involved in protein structure or function but can react with the intracellular environment. Such a reactive Cys occurs in Hb of a widespread European rodent, the bank vole *Clethrionomys glareolus* (Schreber, 1780), for which the ancestral serine (Ser) and the derived Cys allele show a clear-cut north-south separation in Britain, with a narrow cline running through northern England [25]. The pattern was originally described by Hall [26] for two electrophoretically detected variants, termed Hb S (slow) and Hb F (fast). By nucleotide sequencing of the complete repertoire of five globin genes encoding the adult α and β subunits in the bank vole, Kotlík and colleagues [25] confirmed that the two Hb types differ only by this single amino acid residue (codon 52) located in exon 2 of the HBB-T1 gene encoding the β-globin and represent geographically partitioned functional variation [25]. Using the TRAP test (total radical-trapping antioxidant potential) to measure the antioxidant capacity of the erythrocytes the authors demonstrated that voles carrying Hb F, a variant occurring predominantly in southern Britain, showed significantly increased erythrocyte resistance to a free-radical attack compared to voles carrying Hb S, a variant occurring primarily in northern Britain [25]. The mechanistic basis indicated by molecular modelling [25] is that the exposed and negatively charged side chain sulphur atom makes the β52Cys a highly reactive residue similar to highly reactive Cys residues in Hbs of some other rodents [27,28]. There is evidence that the highly reactive Cys in rat and mouse Hb take part in the regeneration of the reduced (active) form of the major intracellular antioxidant glutathione (GSH) through a thiol-disulphide exchange reaction with the oxidized (inactive) glutathione disulphide (GSSG), which releases a molecule of GSH [22,27]. Furthermore, Hb thiols are likely involved in direct reactive oxygen species (ROS) scavenging owing to their high intracellular concentration and the ability to react with ROS directly [27]. 

Because the rate of ROS production markedly increases during energetically demanding physiological states, such as muscular activity, increased growth rate or reproduction, or under thermal stress [29], Hb F would likely be advantageous under a multitude of ecological conditions [30]. It was therefore hypothesised [25,26] that the geographic pattern displayed by Hb S and Hb F in Britain was a result of natural selection by geographical variation in environmentally induced oxidative stress (a disturbance in the balance between generation of ROS and their elimination by antioxidant defence mechanisms).

The Cys allele most plausibly arrived to Britain from continental Europe at the beginning of the Holocene when the Ser allele was already present in Britain and spread at the expense of the Ser allele through the southern portion of the island [25]. Here, we describe the spatial pattern of the Ser/Cys polymorphism throughout the continental range of the bank vole and test for the geographic correlation between environmental variables and the Cys allele frequency expected if the polymorphism was under spatially heterogeneous environmental selection [5,31]. Although they provide no definitive demonstration of direct causal effects, correlation analyses are useful to generate hypotheses about underlying selective forces shaping the genetic patterns when there is little previous information to allow for identification of specific hypotheses a priori [32]. Caution is required for using correlation analyses with geographically structured data as they can yield spurious correlations if the geographical structure (e.g., due to shared demographic history) of the dependent variable (e.g., allele frequency) accidentally matches the spatial structure of the explanatory variable [33]. To overcome this limitation, we apply a principal component analysis (PCA) to reduce dimensionality and thus the variable non-independence, as well as a multivariate modelling approach allowing for correction for population structure [33], taking advantage of the detailed knowledge of the range-wide patterns of bank vole phylogeography available from previous studies [34,35,36,37]. 

For genotyping the polymorphic variants, we designed a pyrosequencing assay for the PyroMark system (Qiagen, Hilden, Germany). It has been previously shown that the same pair of Ser and Cys alleles also segregate at the second, low-expression gene copy, HBB-T2 [25], a pattern that could possibly be attributed to a history of interparalog gene conversion [38,39]. We therefore designed two separate assays to score the polymorphism in HBB-T1 and HBB-T2. Furthermore, we sequenced representative Ser and Cys haplotypes from each gene to assess gene conversion as a potential mechanism for altering the function of Hb as antioxidant by transferring the alleles between the high- and low-expressed genes, of relevance to the adaptive response to end-glacial colonization by the bank vole. Generalising from the different pieces of our study, we argue that it is possible for differentially expressed gene duplicates segregating the same beneficial polymorphism to have a role in population adaptation to new or changing environments.

## 2. Materials and Methods 

### 2.1. Samples

We analysed a total of 518 voles from 136 sampling sites across continental Europe. Samples were selected to cover the bank vole distribution and to include representatives of the different intraspecific clades previously inferred from mitochondrial DNA (mtDNA) phylogeography [34,35,36,37]. Small sample sizes from some sites precluded analysis of all 136 sites. Therefore, geographically close sites were pooled together, resulting in 72 populations (Table S1). Total genomic DNA was isolated from ethanol preserved tissues (liver, spleen or toe, tail or ear clips) using the Qiagen (Valencia, CA, USA) DNeasy Blood and Tissue Kit. For Britain, available data for a total of 145 bank voles from 12 localities along a north-south transect [25] were included in the analysis of genotype-environment correlation. 

### 2.2. Genotyping

For fast and accurate genotyping of the codon 52 polymorphic site a pyrosequencing method using PyroMark Q24 (Qiagen) was applied. The PyroMark Q24 Assay Design Software v 2.0 (Qiagen) was used to design two separate assays to type the C to G nucleotide polymorphism changing codon 52 from TCC (encoding Ser) to TGC (encoding Cys) in HBB-T1 and in HBB-T2, respectively. Each assay used two amplification primers and a sequencing primer. To make the assays gene specific, a reverse amplification primer was in each case designed within the 3′ untranslated region (UTR), which shows consistent differences between the two genes [25]. The PyroMark Q24 protocol was modified to increase the volume of Streptavidin Sepharose beads in the immobilisation step from the recommended 1 μL to 3 μL, which helped to ensure efficient binding of the amplicons longer than 900 bp (Table S2), which is approximately double the recommended length.

The assays were used to genotype both genes in 417 voles and HBB-T1 in an additional 49 voles. The HBB-T2 genotypes for those 49 voles and genotypes at both genes for a further 52 voles were obtained by Sanger sequencing using the sequencing primers BT1F1 (5′ ACAYTTGCTTCTGACATAGT 3′) for HBB-T1 and HBB10U19 (5′ ATGCACACCCTGGAATTGG 3′) for HBB-T2. For the complete list of primers see Table S2.

Allele frequencies were calculated using GENEPOP v 4.2 [40] and frequency surfaces calculated using the inverse distance weighted (IDW) interpolation method in 3D Analyst tools in ArcGIS v 10.2 (ESRI, Redlands, CA, USA). The linkage phase of the Ser and Cys alleles between HBB-T1 and HBB-T2 was inferred by the PHASE algorithm, a coalescent-based Bayesian method [41,42], as implemented in the DnaSP software v 6.10.01 [43].

The genotype frequencies were tested for Hardy-Weinberg equilibrium (HWE) in GENEPOP v 4.2 [40] using an exact HWE test [44]. Three variants of the exact test were used, one to test for any deviation from HWE in the population and two others that specifically test either heterozygote excess or deficit [45]. The significance level (α) was set at 0.05 and a Bonferroni correction applied for the number of populations tested. Association between Hb genotype and the intraspecific phylogeographic clade was tested in voles with data available for both Hb and mtDNA [25,36], which totalled 391 voles for HBB-T1 and 386 voles for HBB-T2. The allelic disequilibria measuring the non-random association between alleles at the nuclear locus and mtDNA were calculated using the CND package [46,47]. Statistical significance with α set at 0.05 was assessed using the asymptotic test [47].

### 2.3. Testing for Genotype-Environment Correlation

A set of 19 temperature and rainfall variables (Bioclim dataset) at a 30 s resolution was downloaded from the WorldClim database ([48]; http://worldclim.org; for the list of the variables and the abbreviations see Table S3). Values were extracted for each site (including the British populations but excluding the Irish that is the result of human introduction [49]) where HBB-T1 genotype data were available (136 sites) using the ArcGIS v 10.2 (ESRI) Spatial Analyst toolbox. Latitude, longitude and altitude were included as additional variables, using the WorldClim altitude data for sites where field-recorded GPS altitude was not available. A weighted average was used for population samples comprising more than one sampling point.

As a first step to reveal possible association between β-globin polymorphism and environment, principal component analysis (PCA) of the environmental data was performed in Statistica 10 (StatSoft Inc., Tulsa, OK, USA) to reduce dimensionality and the variable non-independence. The correlations of the principal components with the Cys allele frequency, latitude, longitude and altitude were then assessed by calculating the Spearman’s rho correlation coefficient (α = 0.05).

A more rigorous spatial analysis was performed with the Samβada program v 0.5.1, which uses logistic regression to model the probability of occurrence of an allele given the environmental conditions at the sampling locations [50]. First, a univariate analysis was performed, where a model (M) containing a single environmental (explanatory) variable and the Cys allele as the binary response variable is compared to a null model (M0) in which the probability of presence of the genotype equals to its frequency [33]. Significance of M is assessed with the log-likelihood ratio (G) and Wald tests, scores of which follow a chi-square distribution that is used to derive the *p*-value of the tests. A Bonferroni correction is applied for multiple comparisons. Then, a multivariate approach was applied, where models with *q* explanatory variables are compared to simpler, *q*−1 ‘parent’ models and the significance of the increase in likelihood is evaluated by a likelihood ratio test. The Wald test assessing whether each of the q regression coefficients β is significantly different from zero was also adopted to attach significance values to the parameters in multivariate models. To account for the effect of population structure, the probability of belonging to the Western mtDNA phylogroup (zero or one, see Results) was added as an additional explanatory variable [33]. Multivariate models of up to four parameters were calculated, with α set at 0.01. This approach enabled us to take into account the historically determined population structure and assess whether adding an environmental variable to the model provides a better explanation of the Cys allele distribution than that based on population structure alone [33]. The datasets for continental Europe and Britain were analysed separately as well as in combination and separate analyses were also performed for mtDNA clades, in which the total Cys allele frequency exceeded 0.15.

### 2.4. Gene Sequencing

Complete gene sequences of HBB-T1 and HBB-T2 (exons and introns, from start to stop codon) representing Ser as well as Cys haplotypes were obtained by Sanger sequencing from 66 voles from the different parts of the species distribution (Table S4). The sequencing followed previously published protocols [25]. For each gene, the haplotype phase between heterozygous sites was inferred by PHASE algorithm as above. For 19 HBB-T1 and 16 HBB-T2 genotypes for which the haplotypes could not be computed with a probability of at least 0.95 or which contained heterozygous indels, the haplotypes were determined experimentally. The amplicons were purified using the QIAquick PCR Purification Kit (Qiagen) and cloned with a Qiagen PCR Cloning plus Kit. Plasmid DNA was isolated from colonies using the QIAprep Spin Miniprep Kit (Qiagen). Six clones containing a fragment of the correct length were sequenced for each amplicon.

Sequences generated in this study were deposited to GenBank (accession numbers MK002878–MK002972).

### 2.5. Sequence Analysis

Alignments of unique haplotypes of each gene were generated with DnaSP software v 6.10.01 [43] and tested for recombination using the HyPhy package accessible through the Datamonkey web (http://www.datamonkey.org./) [51,52]. Phylogenetic trees were constructed for gene segments identified to have distinct evolutionary histories by partitioning the alignments at the estimated breakpoints. Two methods, Single Breakpoint Recombination (SBP) which takes into account only one breakpoint at a time and Genetic Algorithm Recombination Detection (GARD) which considers all possible breakpoints at once, were used. Both methods split the alignment at the position of possible breakpoint(s) and search for the segment-specific phylogenies. The goodness of fit is assessed by the Akaike Information Criterion (AIC) and its small sample correction version (AICc), derived from a maximum likelihood model fit to each segment [53,54]. Maximum likelihood (ML) trees were estimated with Mega 7 software [55] using the best-fit substitution model for a particular dataset chosen by the Bayesian information criterion (BIC).

The haplotypes of HBB-T1 and HBB-T2 were then aligned together and analysed for signatures of inter-paralog gene conversion by the method of Betrán et al. [56] implemented in DnaSP v 6.10.01 and using the GENECONV program v 1.81a [57,58]. The GENECONV analysis was performed with the G-scale parameter (mismatch penalty) set to 0 (no mismatch allowed) and 2 to maximise the chance of detection of recent as well as older conversion events that may be partially masked by subsequent substitutions.

To test for signatures of diversifying selection [14,59] we conducted a sliding window test of silent site diversity within (π; [60]) and divergence between (Dxy; [60]) the Cys and Ser haplotypes from both the HBB-T1 and HBB-T2 genes with DnaSP software v 6.10.01 [43]. Sliding window size was set to 50 bp and step size to 10 bp.

## 3. Results

### 3.1. The Geographic Pattern

**Figure 1:**
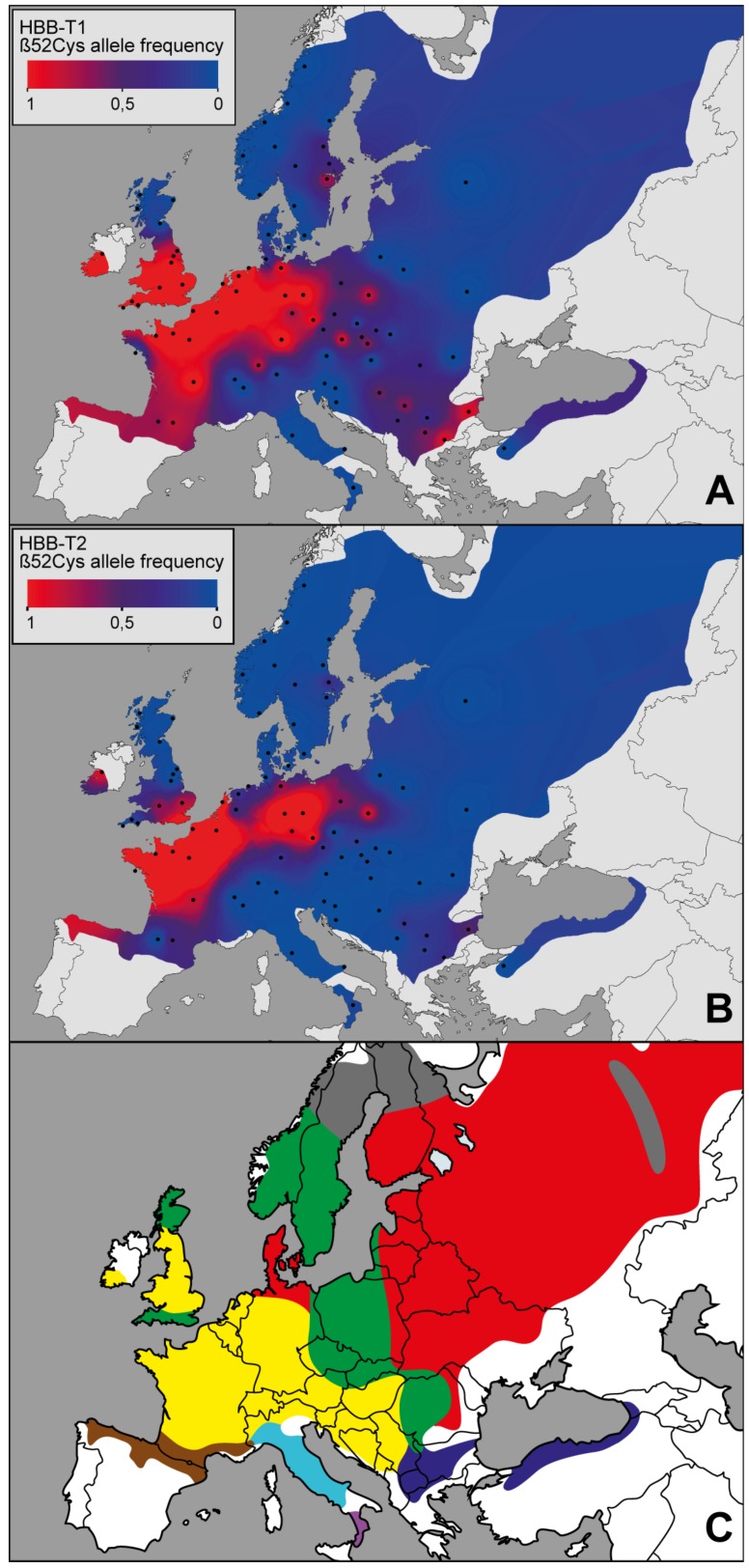
Geographic distribution of the β52Cys allele at HBB-T1 (**A**) and HBB-T2 (**B**) shown as interpolated allele frequency surfaces. Dots represent the location of population samples. Data for Britain were taken from [25]. (**C**) mtDNA lineages distribution modified from [36]. Western lineage in yellow, Carpathian lineage in green, Eastern lineage in red, Balkan lineage in dark blue, Italian lineage in light blue, Calabrian in violet, Pyrenees lineage in brown and introgressed mtDNA from *Clethrionomys rutilus* in grey colour.

From the total of 518 genotyped samples, the genotype at HBB-T1 was determined for 514 voles and at HBB-T2 for 508 voles, with complete two-locus genotypes for both genes obtained for 505 voles. The Cys allele is widely present throughout Europe in both HBB genes but its frequency varies considerably between populations (Figure 1A,B). At HBB-T1, it was found in 45 out of the 72 populations, of which 34 were polymorphic (frequencies between 0.06 and 0.96; see Table S1). At HBB-T2 the Cys allele is geographically more restricted, with six populations fixed and 27 polymorphic (frequencies between 0.05 and 0.94). No deviation from HWE was detected (Bonferroni adjusted *p*-value > 0.05 for all populations).

**Figure 2:**
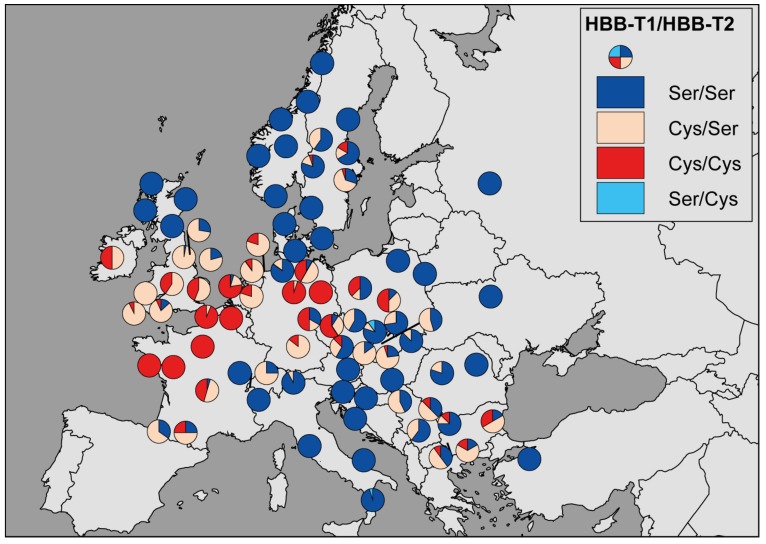
Geographic distribution of the two locus HBB-T1/HBB-T2 haplotypes.

The linkage phase of the codon 52 polymorphic site between the two HBB genes could be resolved unambiguously for all voles (posterior probability of 0.99–1.0). All four possible two-locus (HBB-T1/HBB-T2) haplotypes were detected. However, the haplotype Ser/Cys was only present in two voles from the Czech Republic and one vole from southern Italy and only in heterozygous state SerSer/SerCys (*n* = 3; frequency 0.003). The other three haplotypes were present in high frequencies throughout Europe (Figure 2), with haplotype Ser/Ser being the most frequent (*n* = 590; 0.58), followed by Cys/Ser (*n* = 233; 0.23) and Cys/Cys (*n* = 184; 0.18). Therefore, due to the rarity and restricted pattern of haplotype Ser/Cys (Figure 2), the distribution of the Cys allele at HBB-T2 is governed by the distribution of haplotype Cys/Cys, while the distribution of the Cys allele at HBB-T1 is determined by the combined distribution of haplotypes Cys/Ser and Cys/Cys (Figure 2).

The geographical pattern of the Cys allele at HBB-T1 closely matches the distribution of the Western mtDNA lineage of the bank vole in Europe ([35,36,37], Figure 1C), with the Cys/Cys haplotype showing more restricted distribution in the western part of the continent than Cys/Ser (Figure 2). The analysis of the cytonuclear disequilibria confirmed a significant positive association of the Cys allele and the Western lineage for both genes, while the associations with the Eastern and Carpathian lineages were significantly negative (Table S5), as were the associations (HBB-T1 only) with three other mtDNA lineages, Italian, Gargano and Calabrian.

### 3.2. The Genotype-Environment Correlation

For continental Europe, the first four principal components (PCs) accounted for 89% of the total variance of the original 19 bioclimatic variables (see Table S6). A Spearman correlation test revealed a modest negative correlation between the Cys allele and PC2 (rho = −0.4, *p* < 0.001), PC4 (rho = −0.37, *p* < 0.01), longitude (rho = −0.33, *p* < 0.01) and altitude (rho = −0.24, *p* < 0.05) (see Table S7). PC2 explains 29% of the original variance and the variables with high loadings (above 0.5) on this component include Tseason, TArange and Pseason with positive loadings and MeanTcoldQ, MinTcold, AMT, MeanTdryQ and Isotherm with negative loadings. PC4 explains 6.8% of the variance and contains only one variable with high positive loading, Pseason.

The univariate analysis with Samβada detected 20 out of the 23 models as significant according to the Wald score, including the model with population structure as the explanatory variable (Table 1 and Table S8). In bivariate analysis, 88 out of 253 possible models were significantly better than their univariate parents and eight of the significant models contained population structure as one of the variables (Table S9). Other explanatory variables in the significant models were AP, PcoldQ, PwetQ, Pwet, Pdry, PdryQ, PwarmQ and MeanTwetQ (Table 1 and Table S9). Analysis of trivariate models revealed 137 out of 1770 possible models as significantly better than their bivariate parents; four of these 137 models included population structure among their explanatory variables along with Pseason and PdryQ, Pseason and PcoldQ, Tseason and AP, Tseason (for details see Table 1 and Table S10). Out of the 8835 models with four explanatory variables, 137 were significant but none contained population structure (Table S11). 

Considering Britain on its own, the first two principal components explain 89% of the total variance (see Table S6). Only PC2, accounting for 24% of the variance, shows strong significant positive correlation with the Cys allele (r = 0.82, *p* < 0.01; see Table S7). Among the variables with high positive loading are AMT, MeanTcoldQ, MeanTwarmQ and MinTcold and there is only one variable with high negative loading, Isotherm. In addition, there is a strong negative correlation between the Cys allele and latitude (r = −0.88, *p* < 0.001 (Table S7)). The logistic regression identified 16 out of the 23 possible univariate models as significant but not the model with population structure (Table S12). In the bivariate analysis, on the other hand, 35 out of 253 possible models were significant including a model with population structure and Isotherm as explanatory variables (Table S13). In the trivariate analysis the model containing population structure, Long and TDrange was the only significant one (Table S14). 

For the combined British and continental European dataset, the first four principal components explain 90% of the variance (Table S6). There is a significant negative correlation of the Cys allele with PC2 (r = −0.51, *p* < 0.000001) and PC4 (r = −0.25, *p* < 0.05) and also with longitude (r = −0.33, *p* < 0.01) and altitude (r = −0.26, *p* < 0.05; see Table S7). PC2 explains 29.1% of the variance and contains the same eight variables as in the analysis of continental Europe plus an additional variable with high positive loading, PwarmQ. PC4 explains 7% of the variance and consist of only one variable with high positive loading, Pseason (Table S7). The univariate logistic regression analysis detected 20 out of 23 possible models as significant, including ones incorporating population structure (Table S15). In the bivariate analysis, 111 models were significant and 11 of these contained population structure (Table S16). Other explanatory variables in the significant models were PdryQ, Pdry, PwarmQ, AP, PcoldQ, Elevation, MinTcold, MeanTcoldQ, Pwet, PwetQ and MeanTwarm (Table S16). Among 188 significant trivariate models, 12 contained population structure together with the following pairs of explanatory variables: TArange and PdryQ, TArange and Pdry, Tseason and Pdry, AP and PcoldQ, Tseason and PdryQ, TArange and MeanTwetQ, Long and Pdry, Long and PdryQ, MeanTdry and AP, TArange and MeanTwarmQ, MeanTdryQ and PdryQ, MeanTdryQ and PcoldQ (Table S17). There was no model with population structure among the 133 significant models containing four explanatory variables (Table S18).

Samβada analyses of the Western clade detected 15 significant univariate models and for the Carpathian clade 6 univariate models were detected as significant (Table S19). In the bivariate analysis 20 significant models were identified for the Western clade and none for the Carpathian clade (Table S19). Trivariate analysis detected one model as significant for the Western clade and none for the Carpathian clade (Table S19). 

### 3.3. The Bidirectional Gene Conversion

**Figure 3:**
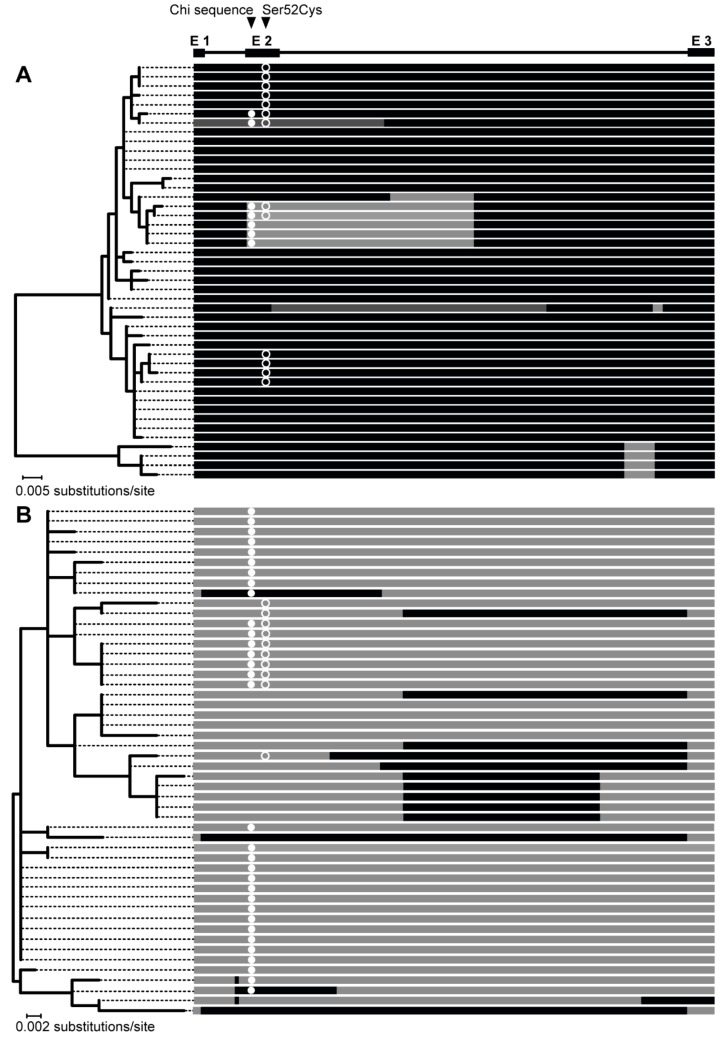
Maximum likelihood (ML) phylogenies based on the HBB-T1 (**A**) and HBB-T2 (**B**) alignments to the left of the recombination breakpoints identified by SBP. Haplotypes are represented by whole gene sequences with mapped conversion tracts. In HBB-T1, tracts identified by the method of Betrán et al. [56] are in light grey, tracts identified by GENECONV are in dark grey. The locations of the β-globin polymorphic site 52 and of the Chi sequence (see Section 4.2.) are depicted by arrows. Exons are marked by rectangles above the alignment.

Complete Sanger sequences of both HBB-T1 and HBB-T2 were obtained for 57 voles, the HBB-T1 sequence only was obtained for 6 additional voles and the HBB-T2 sequence for 3 additional voles, resulting in a total of 246 phased haploid sequences (two sequences per individual, 126 and 120 sequences per gene, respectively). The presence of recombination in HBB-T1 and HBB-T2 was tested for 45 and 50 unique haplotypes identified among the sequences of each gene, respectively (Table S20). The SBP method identified one breakpoint in HBB-T1 at alignment site 521 (according to AIC, ΔAIC = 25.59 between single versus two tree model) and one breakpoint in HBB-T2 at site 329 (according to AIC and cAIC, ΔAIC = 79.34 and corrected ΔAIC = 17.34). The GARD method did not infer any additional recombination events. In the phylogenetic tree constructed for the left (5′) HBB-T1 segment which contains the codon 52 polymorphic site (264), haplotypes with the Cys allele do not form a clade but are split into three groups of seven, two and four haplotypes, which are located in different parts of the tree and are interspersed with haplotypes containing the Ser allele (Figure 3A and Figure S1). In contrast, in the phylogeny inferred for the left segment of HBB-T2, the 10 haplotypes containing the Cys allele form a single clade, with the exception of a haplotype (Hap 77) from southern Italy (Figure 3B and Figure S2). The clustering into the three groups is preserved in a phylogeny of the HBB-T1 segment right of the breakpoint (Figure S3), while in the tree for the right HBB-T2 segment, Cys allele haplotypes are scattered all over the phylogeny (Figure S4).

A total of 28 interparalog gene conversion tracts of various lengths (2–893 bp) were detected by the method of Betrán et al. [56]. Of these, 11 tracts were present in HBB-T1 and 17 in HBB-T2. The GENECONV [57,58] analysis identified additional two tracts in HBB-T1 (370 and 580 bp). It should be noted that DnaSP reports the tracts as bounded by the outermost converted sites (i.e., minimal conversion tracts) while GENECONV delimits the tracts by closest unconverted discriminant sites (i.e., maximal conversion tracts). Most converted haplotypes had a single gene conversion tract, except one HBB-T1 and one HBB-T2 haplotype which had two conversion tracts each (Figure 3, for details see Table S20).

In 10 haplotypes, the conversion tract spanned exon 2 containing the polymorphic codon 52 (sites 263–265) (Figure 3A,B). Six of these are HBB-T1 haplotypes (five with tracts at alignment sites 208–664 and one at sites 1–580) and four are HBB-T2 haplotypes (two with tracts at sites 66–959, one at sites 182–514 and one at sites 66–579). All four HBB-T2 haplotypes with exon 2 converted by HBB-T1 contain a Ser allele while three of the six HBB-T1 haplotypes with exon 2 converted by HBB-T2 contain a Ser allele and three Cys alleles (Figure 3A). The haplotypes containing a Cys allele have conversion tracts of different length and they each come from another geographic region: one from Serbia (Hap 13; tract 1–580), one from France (Hap 24; tract 208–664) and one from Sweden (Hap 27; tract 208–664).

**Figure 4:**
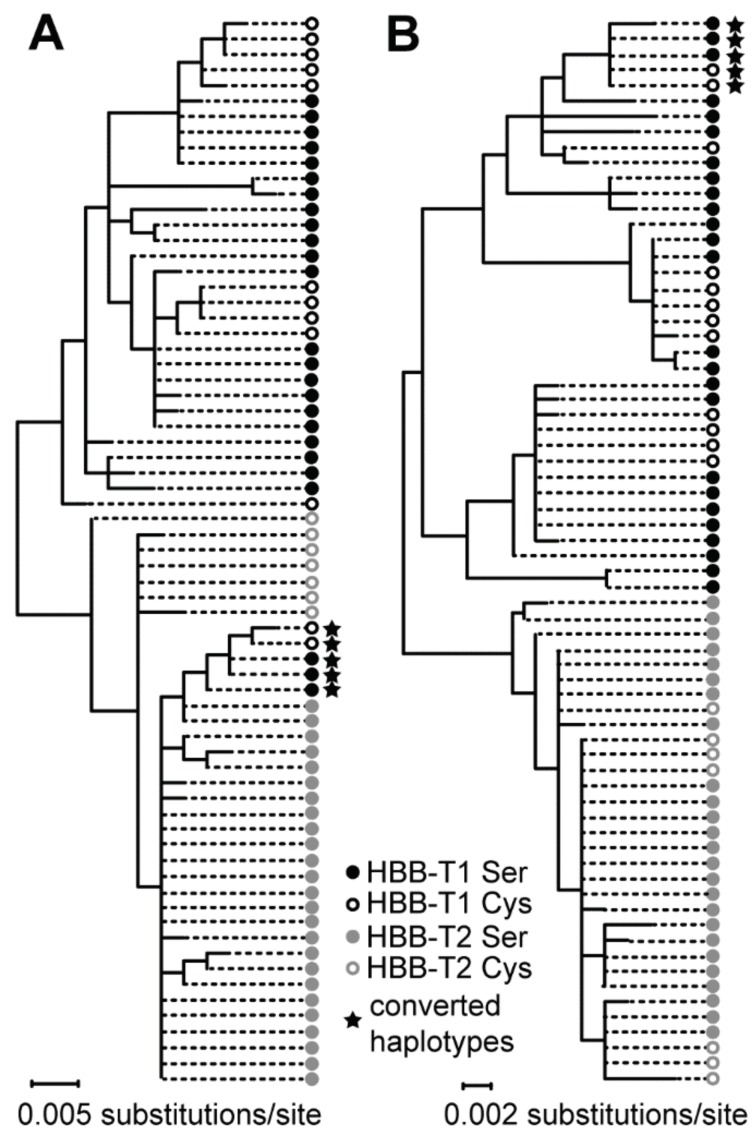
Schematic representation of ML trees for both HBB-T1 and HBB-T2 analysed together, representing (**A**) the converted segment of the gene spanning the sites 208–664 and (**B**) the remaining two unconverted segments of the gene (concatenated sites 1–207 and 665–1128). Haplotypes containing converted tracts in other regions were excluded.

Phylogenetic analysis of the alignment segment spanning the conversion tract at sites 208–664 clustered the five converted HBB-T1 haplotypes into a cluster with HBB-T2 haplotypes (Figure 4 and Figure S5), which supports their origin by gene conversion.

The homogenizing effect of gene conversion around exon 2 was supported by the sliding window analysis showing a sharp drop in both sequence diversity (π) and divergence (Dxy) centred on exon 2 and the adjacent intron region (Figure S6).

## 4. Discussion

### 4.1. Is the Polymorphism under Environmental Selection?

It has been demonstrated through in vitro experiments that bank vole Hb F increases erythrocyte resistance to oxidative stress and this effect has been linked to the reactivity of β52Cys encoded on HBB-T1, the major β-globin gene in bank vole [25]. Levels of oxidative stress are tightly linked to environmental variation [61,62] and it was therefore proposed that the clear-cut geographic pattern displayed by the Ser and Cys alleles in Britain may reflect local adaptation to different environmental conditions [25,26]. Here, we mapped the distribution of the Cys allele throughout Europe and applied a correlative approach allowing correction for neutral population structure to test for the association between abiotic environmental variables and the distribution of Hb F.

The genotyping data demonstrate that in continental Europe the distribution of the Cys allele shows strong geographic patterning with a high frequency in western Europe and a decline northwards, southwards and eastwards (Figure 1A,B). This distribution closely matches the Western mtDNA clade of the bank vole (Figure 1C), which is consistent with the observation that in Britain the Cys allele occurs in the southern part of the island occupied by bank voles carrying the Western mtDNA clade [36,63]. This is in agreement with the scenario that the Cys allele arrived to Britain with the second wave of colonization at the end of the last glaciation and then spread at the expense of the Ser allele that was already present in Britain at that time as a result of an earlier colonization [25]. Therefore, the bank vole population history has clearly been important in shaping the dispersal of the Cys allele. We found, however, significant association between the environmental variables and the distribution of the Cys allele in Britain as well as in continental Europe, by both PCA-correlation test and by logistic regression modelling with Samβada. Importantly, the association remained significant after inclusion of a correction for population structure in the Samβada models and also by modelling performed separately for the populations of, respectively, the Western and Carpathian mtDNA clades (the major units of the historical population structure of the bank vole; [36]). Therefore, there is strong evidence that environmental variables have a significant predictive value on the genotype at the β52 site even when the population history is taken into account (Table 1).

However, spurious associations between allele frequency and environmental variables could be produced not only by shared history but also by nonadaptive processes such as allele surfing (spread of a random allele due to its association with the front of a wave of population expansion), for example when ecological gradients align with the direction of population expansion [64]. While postglacial expansion likely created opportunities for allele surfing, we consider it as an unlikely explanation here because the Cys allele shows areas of high frequency in various parts of Europe colonised from different glacial refugia (signified by different mtDNA clades) (Figure 1A) and the probability of the same allele at the same locus to surf along multiple expansions should be very small.

Among the variables consistently showing up in the significant models were total annual precipitation (AP), total precipitation during the driest three months of the year (PdryQ) and total precipitation during the coldest three months of the year (PcoldQ), with the Cys allele being present in areas with lower maximum values of these variables. We acknowledge that correlation does not necessarily mean causality per se and that the relationship between environment and oxidative stress is very complex. Therefore multiple abiotic factors including the annual trends and seasonal extremes in water availability and temperature may have either a direct impact on bank vole physiology (e.g., via water or thermal stress; [65]), or a more indirect impact on fitness through, for instance, habitat availability and quality, or abundance and quality of food. The latter may, in turn, affect, for example, population density and cyclicity, or the diversity and abundance of pathogens and parasites and therefore represent potential selective forces acting on a longer time scale. Further testing of differences between the bearers of the two Hb variants on cellular and organismal level will be necessary to evaluate the particular effect of Hb F on fitness.

In summary, the association between climate variables and the Cys allele found in this study supports the role of the Hb polymorphism in local adaptation in the bank vole. The Cys allele codes for a highly reactive Cys on the major β-globin chain and results in the formation of Hb F responsible for increased erythrocyte resistance to oxidative stress [25]. Therefore, it is likely that the Cys allele would be favoured over the Ser allele in environments selecting for tolerance to increased ROS production. Theory predicts that polymorphism at a locus can be stably maintained by heterogeneous selection when the allele adaptive in one environment is maladaptive in another; otherwise the polymorphism is expected to be eliminated via fixation of the beneficial allele [66,67]. An experimental approach will be needed to elucidate the role of such antagonistic pleiotropy in the maintenance of the bank vole Hb polymorphism but one possibility for a lower relative fitness of the Cys allele in some environments (i.e., those not strongly selecting for ROS tolerance) may be a metabolic cost associated with Hb F synthesis. The majority of Cys available to an organism is utilised for synthesis of glutathione (GSH), the principal thiol-containing metabolite in mammalian cells playing an important role in a multitude of cellular processes such as redox signalling, cell proliferation, differentiation and apoptosis. Therefore, there is likely a trade-off in Cys allocation between GSH and Hb F (e.g., [68]). Additionally, highly-reactive Cys in proteins are prone to a variety of potentially harmful chemical reactions, which might contribute to the fitness cost of the Cys allele, resulting in a reduced fitness in environments where it does not provide strong functional advantage [69].

### 4.2. Gene Conversion as a Possible Function-Altering Mechanism

Although the functional difference between Hb S and Hb F is determined by the genotype at codon 52 of HBB-T1, the same two Ser and Cys alleles segregate at codon 52 in the second gene copy, HBB-T2 [25]. HBB-T2 makes little contribution to β-globin synthesis due to the low expression level [25] but it could affect the Hb function if an HBB-T2 haplotype carrying one allele serves as a donor for gene conversion of an HBB-T1 haplotype carrying the alternate allele. We found evidence of a partial conversion of HBB-T1 by HBB-T2 resulting in six HBB-T1 haplotypes in which exon 2 (and adjacent intron regions) are replaced by exon 2 from HBB-T2 (Figure 3A). The fact that three of these haplotypes carried the Cys allele may be interpreted that the point mutation changing the codon 52 from Ser to Cys occurred in HBB-T2 and was transferred into HBB-T1 by gene conversion. However, an alternative possibility is that the mutation initially occurred in HBB-T1 but was transferred by gene conversion into HBB-T2 and then back into HBB-T1 by subsequent gene conversion event(s) in the opposite direction. Consistent with the latter scenario, there is clear evidence of conversion of exon 2 in HBB-T1 as well in HBB-T2 (Figure 3B). Furthermore, the widespread distribution and high frequency (*n* = 233) of the two-locus (HBB-T1/HBB-T2) haplotype Cys/Ser (Figure 2) strongly supports the origin of the mutation in HBB-T1 rather than in HBB-T2. The haplotype Ser/Cys is very rare (*n* = 3) and is therefore most likely a result of recent recombination [70]. In any case, the gene conversion of exon 2 that transferred the Cys allele from HBB-T2 to HBB-T1 appears to have occurred repeatedly and independently in different populations because the converted haplotypes are placed in two different clades and the inferred conversion tracts have different length in each clade (Figure 3A).

Frequency of gene conversion of certain genomic regions can be increased by the presence of crossover hotspots [71]. We examined the HBB sequences for known crossover hotspot motifs (e.g., [72]) and found an intact Chi site (5′ GCTGGTGG 3′) at nucleotides 4–11 in exon 2, that is, 254 bp upstream of codon 52 (Figure 3A). The Chi site is a well-characterised gene conversion hotspot in prokaryotes [73,74,75], related to locally increased frequency of non-homologous recombination also in a number of mammalian gene families [76,77,78,79]. We found the Chi site on most HBB-T2 haplotypes (Figure 3B) but only on seven HBB-T1 haplotypes, six of which have exon 2 converted by HBB-T2 (Figure 3A). Interestingly, although not found in available sequences of HBB genes of other rodents, a Chi site is found at the exact same location in the human HBB gene, where it has been related to gene conversion as the explanation for the occurrence of a single malaria-protective mutation [80] on multiple haplotype backgrounds [77]. Therefore, the rate at which gene conversion in the bank vole HBB genes involves codon 52 may have been predetermined by the presence of a gene conversion hotspot.

These results suggest some possible interesting adaptive consequences of gene conversion in the bank vole two-locus β-globin system. If, as supported by our analyses, the Cys allele confers selective advantage under certain environmental conditions but not in others, gene conversion between HBB-T1 and HBB-T2 could facilitate local adaptation by restoring two-locus haplotypes that are maladaptive under current local conditions and have been eliminated from the population [81] but which can be adaptive under novel conditions. For instance, the Cys allele can become fixed in HBB-T1 under conditions favouring oxidative stress while HBB-T2 can remain polymorphic for a prolonged period of time because of the relaxed selection pressure due to the low-expression. Widespread coexistence of two-locus haplotypes Cys/Cys and Cys/Ser in southern Britain and western Europe (Figure 2) may therefore be explained by weak selection pressure in HBB-T2 on locally beneficial Cys allele. However, should the local conditions change so that the Cys allele is no longer favoured (the fitness cost outweighs its protective effect), haplotype Ser/Ser created by gene conversion from haplotype Cys/Ser can become more frequent. Similarly, ‘hiding’ in HBB-T2 may enable local survival of the Cys allele under temporarily unfavourable local conditions (Figure 2). Therefore, gene duplication coupled with gene conversion between the differentially expressed gene duplicates may facilitate maintenance of the polymorphism in the bank vole Hb Cys content and allow tuning of the erythrocyte thiol levels in response to the selection pressure imposed by local environment. We suggest that such capacity of adaptive tuning of the erythrocyte antioxidant system may have provided an advantage to bank vole populations carrying the Cys allele during range expansion at the end of the last glaciation [25], as the migrating populations were likely confronted with novel selective pressures or with changes in strength or direction of selection. Therefore, what we describe may represent an example of the complex mutational and selective processes that need to be incorporated into phylogeographic interpretation of end-glacial colonization of moderate/high latitude, that is, an example of what needs to be included in an ‘adaptive phylogeography’ perspective [25]. Furthermore, standing variation as a reservoir for adaptation maintained by various mechanisms, including possibly the one that we describe here, is likely to be highly relevant under the current scenario of climate change when species may need to adapt quickly to rapid environmental change or habitat expansion [82].

## Figures and Tables

**Table 1:** Results of spatial analysis of correlation between the β52Cys allele frequency, population structure and environmental variables for continental Europe, using the Samβada program and showing the 10 best univariate models according to the Wald score and the multivariate models containing population structure; α = 0.01. Population structure was represented by the probability of belonging to the Western lineage (essentially zero or one). A set of 19 temperature and rainfall variables (Bioclim dataset available in the WorldClim database) was used as the environmental variables.

Model	Variable	Variable 2	Variable 3	Log Likelihood	G Score	Wald Score
Univariate	Isotherm ^1^			−307.98	93.63	67.76
	PwetQ ^2^			−304.82	99.94	58.08
	Pwet ^3^			−308.24	93.11	55.01
	AP ^4^			−318.05	73.49	47.24
	MeanTcoldQ ^5^			−330.26	49.07	42.95
	AMT ^6^			−330.70	48.19	41.25
	MinTcold ^7^			−332.83	43.93	39.27
	LONG ^8^			−334.14	41.31	37.01
	Pseason ^9^			−334.78	40.04	36.33
	PopStr ^10^			−255.33	198.93	34.57
Bivariate	PopStr	AP		−221.04	68.57	39.40
	PopStr	PcoldQ ^11^		−230.18	50.29	30.66
	PopStr	PwetQ		−222.44	65.78	29.65
	PopStr	Pwet		−225.44	59.78	29.37
	PopStr	Pdry ^12^		−235.88	38.89	27.09
	PopStr	PdryQ ^13^		−235.97	38.72	26.82
	PopStr	PwarmQ ^14^		−239.11	32.44	25.23
	PopStr	MeanTwetQ ^15^		−243.04	24.58	21.76
Trivariate	PopStr	Pseason	PdryQ	−223.97	24.00	22.21
	PopStr	Pseason	PcoldQ	−217.72	24.93	22.06
	PopStr	Tseason ^16^	AP	−207.47	27.15	21.64
	PopStr	Tseason	PcoldQ	−210.55	39.28	21.16

^1^ Isotherm—isothermality (mean diurnal range/temperature annual range) × 100); ^2^ PwetQ—precipitation of wettest quarter; ^3^ Pwet—precipitation of wettest month; ^4^ AP—annual precipitation; ^5^ MeanTcoldQ—mean temperature of coldest quarter; ^6^ AMT—annual mean temperature; ^7^ MinTcold—minimal temperature of the coldest month; ^8^ LONG—longitude; ^9^ Pseason—precipitation seasonality; ^10^ PopStr—population structure; ^11^ PcoldQ—precipitation of coldest quarter; ^12^ Pdry—precipitation of driest month; ^13^ PdryQ—precipitation of driest quarter; ^14^ PwarmQ—precipitation of warmest quarter; ^15^ MeanTwetQ—mean temperature of wettest quarter; ^16^ Tseason—temperature seasonality (standard deviation × 100).

## Supplementary Materials

The following are available online at http://www.mdpi.com/2073-4425/9/10/492/s1, Table S1: List of the 72 population samples derived from the 136 sampling localities, Table S2: Primers used for pyrosequencing and Sanger sequencing, Table S3: Bioclimatic variables used for Hb genotype–environment analysis as available at http://www.worldclim.org/bioclim and their abbreviations used in text, Table S4: List of samples selected for whole Hb gene sequencing by Sanger sequencing method, Table S5: Allelic cytonuclear disequilibria for Europe (EU) and for the combination Europe and Britain (EU+GB), Table S6: Loadings of variables comprising principal components for the datasets containing data for continental Europe (EU), Britain (GB) and for the combined dataset (EU+GB), Table S7: Spearman’s rho and associated *p*-value for correlation between HBB-T1 52Cys allele frequency and latitude, longitude, altitude and principal components identified in PCA, Tables S8–S18: Results from Samβada analysis for continental Europe (1–4 variables models), Britain (1–3 variables models) and combined (1–4 variables models) datasets, Table S19: Result from Samβada analysis within Western and Carpathian mtDNA clade, 1–3 variables models, Table S20: Alignment of HBB-T1 and HBB-T2 haplotypes, showing variable sites only. Identified gene conversion tracts are shown, Figure S1: Maximum likelihood phylogeny of HBB-T1 haplotypes based on the alignment segment left of the breakpoint at site 521, Figure S2: Maximum likelihood phylogeny of HBB-T2 haplotypes based on the alignment segment left of the breakpoint at site 329, Figure S3: Maximum likelihood phylogeny of HBB-T1 haplotypes based on the alignment segment right of the breakpoint at site 521, Figure S4: Maximum likelihood phylogeny of HBB-T2 haplotypes based on the alignment segment right of the breakpoint at site 329, Figure S5: Maximum likelihood phylogeny for both genes representing the converted gene segment spanning sites 208–664, Figure S6: Results of a sliding window test of silent site diversity within (π) and between (Dxy) the Cys and Ser haplotypes.
