# Supplementary material for: Playing Hide-and-Seek in Beta-Globin Genes: Gene Conversion Transferring a Beneficial Mutation between Differentially Expressed Gene Duplicates
Source: Genes (Basel). 2018 Oct 12;9(10):492. doi: 10.3390/genes9100492 (PMC6209878; doi:10.3390/genes9100492)
Supplement: Supplementary file 1 [file genes-09-00492-s001.zip › Supporting_information.docx]

**Supporting information:**

Table S1. List of the 72 population samples derived from the 136 sampling localities.

| Population | Population latitude | Population longitude | Nr. of samples in population | HBB-T1 Cys frequency | HBB-T2 Cys frequency | Country | Locality latitude | Locality longitude | Locality altitude (m) | Nr. of samples |
| --- | --- | --- | --- | --- | --- | --- | --- | --- | --- | --- |
| AT1 | 47.85 | 15.05 | 10 | 0.000 | 0.000 | Austria | 47.85 | 15.05 | 880* | 10 |
| BEL1 | 50.63 | 4.18 | 5 | 1.000 | 1.000 | Belgium | 50.63 | 4.18 | 99 | 5 |
| BGR1 | 43.10 | 23.40 | 4 | 0.250 | 0.125 | Bulgaria | 43.10 | 23.40 | 618 | 4 |
| BGR2 | 42.20 | 23.00 | 5 | 0.600 | 0.100 | Bulgaria | 42.20 | 23.00 | 766 | 5 |
| BGR3 | 41.50 | 24.50 | 3 | 0.833 | 0.167 | Bulgaria | 41.50 | 24.50 | 1267 | 3 |
| BGR4 | 42.91 | 27.05 | 3 | 0.833 | 0.333 | Bulgaria | 42.93 | 27.65 | 197* | 2 |
|  |  |  |  |  |  | Bulgaria | 42.88 | 25.86 | 618* | 1 |
| CZAT | 48.84 | 16.66 | 11 | 0.818 | 0.000 | Austria | 48.37 | 16.23 | 170* | 1 |
|  |  |  |  |  |  | Czech Republic | 48.97 | 16.60 | 166* | 5 |
|  |  |  |  |  |  | Czech Republic | 48.80 | 16.80 | 157* | 5 |
| CZE1 | 50.23 | 13.95 | 11 | 0.909 | 0.591 | Czech Republic | 50.21 | 13.90 | 378* | 10 |
|  |  |  |  |  |  | Czech Republic | 50.42 | 14.47 | 260 | 1 |
| CZE2 | 49.57 | 14.90 | 12 | 0.417 | 0.125 | Czech Republic | 49.80 | 18.22 | 548 | 11 |
|  |  |  |  |  |  | Czech Republic | 48.98 | 14.43 | 475* | 1 |
| CZE3 | 50.50 | 16.12 | 6 | 0.417 | 0.000 | Czech Republic | 50.50 | 16.12 | 500 | 6 |
| CZE4 | 49.75 | 18.30 | 10 | 0.100 | 0.100 | Czech Republic | 49.70 | 18.38 | 318* | 5 |
|  |  |  |  |  |  | Czech Republic | 49.80 | 18.22 | 201* | 5 |
| CZSK | 48.86 | 18.58 | 11 | 0.545 | 0.000 | Czech Republic | 49.01 | 17.76 | 287* | 1 |
|  |  |  |  |  |  | Slovakia | 48.84 | 18.66 | 282* | 10 |
| DE1 | 53.62 | 7.27 | 10 | 1.000 | 0.200 | Germany | 53.62 | 7.27 | -1 | 10 |
| DE2 | 54.22 | 9.11 | 10 | 0.150 | 0.000 | Germany | 54.22 | 9.11 | 26 | 8 |
|  |  |  |  |  |  | Germany | 54.23 | 9.11 | 1 | 2 |
| DE3 | 53.65 | 10.78 | 12 | 0.917 | 0.417 | Germany | 53.68 | 10.75 | 30 | 10 |
|  |  |  |  |  |  | Germany | 54.21 | 11.07 | 2 | 1 |
|  |  |  |  |  |  | Germany | 52.80 | 10.83 | 86 | 1 |
| DE4 | 51.85 | 11.18 | 10 | 1.000 | 0.950 | Germany | 51.78 | 11.15 | 122 | 9 |
|  |  |  |  |  |  | Germany | 52.50 | 11.46 | 73 | 1 |
| DE5 | 51.86 | 13.06 | 10 | 1.000 | 1.000 | Germany | 51.86 | 13.06 | 96 | 10 |
| DE6 | 50.71 | 11.87 | 3 | 0.667 | 0.500 | Germany | 50.17 | 11.38 | 470 | 1 |
|  |  |  |  |  |  | Germany | 50.97 | 12.14 | 293 | 1 |
|  |  |  |  |  |  | Germany | 50.99 | 12.08 | 293 | 1 |
| DE7 | 49.02 | 10.73 | 7 | 1.000 | 0.143 | Germany | 49.02 | 10.73 | 542 | 7 |
| DNK1 | 55.80 | 9.41 | 5 | 0.000 | 0.000 | Denmark | 55.63 | 8.48 | 19 | 2 |
|  |  |  |  |  |  | Denmark | 55.27 | 8.94 | 19 | 1 |
|  |  |  |  |  |  | Denmark | 56.23 | 10.57 | 57 | 2 |
| DNK2 | 54.96 | 11.63 | 7 | 0.000 | 0.000 | Denmark | 55.73 | 11.92 | 17 | 1 |
|  |  |  |  |  |  | Denmark | 54.87 | 11.71 | 5 | 2 |
|  |  |  |  |  |  | Denmark | 54.83 | 11.67 | 4 | 2 |
|  |  |  |  |  |  | Denmark | 54.81 | 11.36 | 8 | 2 |
| FRA1 | 47.34 | -3.17 | 1 | - | 1.000 | France | 47.34 | -3.17 |  | 1 |
| FRA2 | 48.60 | -1.50 | 4 | 1.000 | 1.000 | France | 48.60 | -1.50 | 15 | 4 |
|  |  |  |  |  |  |  |  |  |  |  |
| Population | Population latitude | Population longitude | Nr. of samples in population | HBB-T1 Cys frequency | HBB-T2 Cys frequency | Country | Locality latitude | Locality longitude | Locality altitude (m) | Nr. of samples |
| FRA3 | 48.97 | 0.13 | 5 | 1.000 | 1.000 | France | 48.92 | 0.20 | 99 | 4 |
|  |  |  |  |  |  | France | 49.17 | -0.15 | 1 | 1 |
| FRA4 | 50.53 | 1.78 | 9 | 0.889 | 0.938 | France | 50.08 | 1.57 | 71 | 1 |
|  |  |  |  |  |  | France | 50.17 | 1.63 | 27 | 3 |
|  |  |  |  |  |  | France | 50.87 | 1.82 | 26 | 3 |
|  |  |  |  |  |  | France | 50.75 | 2.25 | 14 | 1 |
|  |  |  |  |  |  | France | 50.83 | 1.87 | 109 | 1 |
| FRA5 | 48.71 | 1.76 | 8 | 1.000 | 1.000 | France | 48.55 | 3.40 | 158 | 1 |
|  |  |  |  |  |  | France | 48.75 | 1.55 | 136 | 2 |
|  |  |  |  |  |  | France | 48.72 | 1.52 | 134 | 5 |
| FRA6 | 46.04 | 2.60 | 11 | 0.955 | 0.455 | France | 45.70 | 2.13 | 823 | 1 |
|  |  |  |  |  |  | France | 46.04 | 2.65 | 658 | 4 |
|  |  |  |  |  |  | France | 46.14 | 2.73 | 598 | 2 |
|  |  |  |  |  |  | France | 46.00 | 2.55 | 569 | 2 |
|  |  |  |  |  |  | France | 46.17 | 2.63 | 481 | 2 |
| FRA7 | 43.25 | -0.12 | 10 | 0.650 | 0.000 | France | 43.25 | -0.12 | 402 | 10 |
| FRA7.2 | 43.30 | 1.17 | 4 | 0.750 | 0.250 | France | 43.30 | 1.17 | 221 | 4 |
| HR1 | 45.74 | 15.65 | 6 | 0.000 | 0.000 | Croatia | 45.74 | 15.65 | 443* | 6 |
| HR2 | 44.84 | 15.62 | 10 | 0.000 | 0.000 | Croatia | 44.83 | 15.62 | 643* | 2 |
|  |  |  |  |  |  | Croatia | 44.85 | 15.62 | 570* | 1 |
|  |  |  |  |  |  | Croatia | 44.84 | 15.62 | 770 | 7 |
| HU1 | 47.30 | 19.22 | 7 | 0.000 | 0.000 | Hungary | 47.30 | 19.22 | 102* | 7 |
| CHE1 | 46.38 | 6.35 | 9 | 0.056 | 0.000 | Switzerland | 46.20 | 6.17 | 425 | 4 |
|  |  |  |  |  |  | Switzerland | 46.52 | 6.50 | 400 | 5 |
| CHE2 | 45.90 | 7.20 | 6 | 0.000 | 0.000 | Switzerland | 45.90 | 7.20 | 2087 | 6 |
| CHE3 | 47.37 | 8.55 | 2 | 0.750 | 0.000 | Switzerland | 47.37 | 8.55 | 424 | 2 |
| CHEATIT | 46.78 | 10.27 | 13 | 0.077 | 0.000 | Switzerland | 46.78 | 10.18 | 1450* | 10 |
|  |  |  |  |  |  | Italy | 46.07 | 11.11 | 193 | 1 |
|  |  |  |  |  |  | Austria | 47.37 | 9.97 | 745* | 1 |
|  |  |  |  |  |  | Austria | 46.92 | 10.60 | 1450* | 1 |
| IRL1 | 53.11 | -8.60 | 2 | 1.000 | 0.500 | Ireland | 53.08 | -8.23 | 47 | 1 |
|  |  |  |  |  |  | Ireland | 53.13 | -8.97 | 14 | 1 |
| IT1 | 42.87 | 11.59 | 6 | 0.000 | 0.000 | Italy | 42.52 | 12.12 | 329 | 3 |
|  |  |  |  |  |  | Italy | 43.22 | 11.07 | 438 | 3 |
| IT2 | 41.82 | 16.01 | 4 | 0.000 | 0.000 | Italy | 41.82 | 16.01 | 766 | 4 |
| IT3 | 39.35 | 16.45 | 10 | 0.000 | 0.050 | Italy | 39.30 | 16.12 | 1021 | 1 |
|  |  |  |  |  |  | Italy | 39.35 | 16.49 | 1176 | 9 |
| NLD1 | 52.50 | 4.57 | 11 | 0.955 | 0.773 | Netherlands | 52.80 | 4.70 | -4* | 5 |
|  |  |  |  |  |  | Netherlands | 52.25 | 4.47 | -21* | 6 |
| NLD2 | 53.07 | 6.24 | 5 | 1.000 | 0.100 | Netherlands | 53.07 | 6.24 | 27* | 5 |
| NLD3 | 52.07 | 6.08 | 5 | 1.000 | 0.200 | Netherlands | 52.07 | 6.08 | 79* | 5 |
| NOR1 | 66.42 | 14.77 | 1 | 0.000 | 0.000 | Norway | 66.42 | 14.77 | 154* | 1 |
| NOR2 | 64.15 | 11.97 | 6 | 0.000 | 0.000 | Norway | 64.17 | 12.05 | 56* | 2 |
|  |  |  |  |  |  | Norway | 64.14 | 11.93 | 46* | 4 |
| NOR3 | 62.96 | 8.71 | 1 | 0.000 | 0.000 | Norway | 62.96 | 8.71 | 197* | 1 |
| Population | Population latitude | Population longitude | Nr. of samples in population | HBB-T1 Cys frequency | HBB-T2 Cys frequency | Country | Locality latitude | Locality longitude | Locality altitude (m) | Nr. of samples |
| NOR4 | 60.79 | 5.97 | 8 | 0.000 | 0.000 | Norway | 60.79 | 5.97 | 287* | 8 |
| NOR5 | 61.58 | 10.06 | 9 | 0.000 | 0.000 | Norway | 61.58 | 10.06 | 627* | 8 |
|  |  |  |  |  |  | Norway | 60.22 | 10.73 |  | 1 |
| NOR6 | 58.31 | 8.18 | 12 | 0.000 | 0.000 | Norway | 58.98 | 7.66 | 226* | 1 |
|  |  |  |  |  |  | Norway | 58.24 | 8.23 | 115* | 10 |
|  |  |  |  |  |  | Norway | 58.77 | 9.21 |  | 1 |
| PL1 | 52.45 | 17.10 | 4 | 0.500 | 0.375 | Poland | 52.45 | 17.10 | 272* | 4 |
| PL2 | 51.47 | 19.82 | 4 | 0.750 | 0.500 | Poland | 51.47 | 19.82 | 192* | 4 |
| PL3 | 53.80 | 21.65 | 10 | 0.000 | 0.000 | Poland | 53.80 | 21.65 | 145* | 10 |
| PL4 | 52.73 | 23.85 | 10 | 0.000 | 0.000 | Poland | 52.73 | 23.85 | 168 | 10 |
| RO1 | 46.48 | 23.66 | 6 | 0.333 | 0.000 | Romania | 45.30 | 23.83 | 1722 | 1 |
|  |  |  |  |  |  | Romania | 46.72 | 23.62 | 740 | 5 |
| RO2 | 46.55 | 26.83 | 6 | 0.000 | 0.000 | Romania | 46.55 | 26.83 | 284* | 6 |
| RUS1 | 57.15 | 33.10 | 5 | 0.000 | 0.000 | Russia | 57.15 | 33.10 | 203 | 5 |
| SK1 | 48.38 | 19.08 | 11 | 0.682 | 0.045 | Slovakia | 48.26 | 18.99 | 203* | 1 |
|  |  |  |  |  |  | Slovakia | 48.40 | 19.09 | 350* | 10 |
| SK2 | 49.16 | 20.07 | 11 | 0.273 | 0.000 | Slovakia | 49.20 | 19.75 | 1900 | 1 |
|  |  |  |  |  |  | Slovakia | 49.13 | 20.16 | 1114* | 1 |
|  |  |  |  |  |  | Slovakia | 49.15 | 20.16 | 1491* | 4 |
|  |  |  |  |  |  | Slovakia | 49.17 | 19.98 | 1189* | 2 |
|  |  |  |  |  |  | Slovakia | 49.16 | 20.08 | 1531* | 3 |
| SK3 | 48.77 | 21.39 | 9 | 0.125 | 0.000 | Slovakia | 48.84 | 22.18 | 270* | 1 |
|  |  |  |  |  |  | Slovakia | 48.77 | 21.37 | 279 | 7 |
|  |  |  |  |  |  | Slovakia | 48.70 | 20.69 | 611* | 1 |
| SRB1 | 44.09 | 21.64 | 4 | 0.625 | 0.125 | Serbia | 44.09 | 21.64 | 333* | 4 |
| SRB2 | 43.24 | 20.82 | 5 | 0.400 | 0.000 | Serbia | 43.24 | 20.82 | 1148 | 5 |
| SRBHR | 45.16571 | 19.58 | 7 | 0.571 | 0.000 | Croatia | 45.14 | 17.60 | 128* | 1 |
|  |  |  |  |  |  | Serbia | 45.17 | 19.91 | 172* | 6 |
| SVN1 | 46.11 | 14.67 | 6 | 0.000 | 0.000 | Slovenia | 46.11 | 14.67 | 227* | 6 |
| SWE1 | 62.88 | 17.74 | 10 | 0.000 | 0.000 | Sweden | 62.88 | 17.72 | 209 | 5 |
|  |  |  |  |  |  | Sweden | 62.88 | 17.75 | 94 | 5 |
| SWE2 | 60.17 | 12.77 | 10 | 0.200 | 0.050 | Sweden | 60.14 | 12.81 | 159* | 3 |
|  |  |  |  |  |  | Sweden | 60.17 | 12.76 | 188* | 3 |
|  |  |  |  |  |  | Sweden | 60.19 | 12.74 | 300* | 3 |
|  |  |  |  |  |  | Sweden | 60.17 | 12.76 | 208* | 1 |
| SWE3 | 61.30 | 16.55 | 10 | 0.400 | 0.000 | Sweden | 61.15 | 16.81 | 102 | 5 |
|  |  |  |  |  |  | Sweden | 61.46 | 16.28 | 188 | 4 |
|  |  |  |  |  |  | Sweden | 61.45 | 16.29 | 172 | 1 |
| SWE3.2 | 60.18 | 17.09 | 3 | 0.333 | 0.167 | Sweden | 60.21 | 16.98 | 79 | 2 |
|  |  |  |  |  |  | Sweden | 60.09 | 17.32 | 55 | 1 |
| SWE4 | 57.62 | 12.42 | 1 | 0.000 | 0.000 | Sweden | 57.62 | 12.42 | 109* | 1 |
| SWE5 | 59.21 | 16.63 | 10 | 0.700 | 0.050 | Sweden | 59.29 | 16.70 | 54* | 3 |
|  |  |  |  |  |  | Sweden | 59.30 | 16.79 | 70* | 4 |
|  |  |  |  |  |  | Sweden | 59.00 | 16.36 | 72* | 3 |
|  |  |  |  |  |  |  |  |  |  |  |
| SWE6 | 55.70 | 13.47 | 10 | 0.000 | 0.000 | Sweden | 55.71 | 13.49 | 3* | 5 |
|  |  |  |  |  |  | Sweden | 55.69 | 13.46 | 37* | 5 |
| TUR1 | 40.12 | 29.17 | 10 | 0.000 | 0.000 | Turkey | 40.12 | 29.17 | 2002 | 10 |
| UA1 | 50.41 | 29.72 | 6 | 0.000 | 0.000 | Ukraine | 50.33 | 30.47 | 147 | 5 |
|  |  |  |  |  |  |  | 50.80 | 25.95 | 207 | 1 |

*Altitude data obtained by extraction from World Clim dataset at a 30 seconds resolution using ArcGIS v 10.2 (ESRI) Spatial analyst toolbox; altitude data of other localities captured in the field using a GPS (global positioning system) device.

Table S2. Primers used for pyrosequencing and Sanger sequencing. The sequencing primer designed for HBB-T1 pyrosequencing assays was used also for HBB-T2. Sanger sequencing primers were designed by Kotlík *et al.* (2014).

| sequencing method | gene | primer | sequence | type | direction |
| --- | --- | --- | --- | --- | --- |
| pyrosequencing | HBB-T1 | HBB_T1_RB_F19 | 5' GGCTGCTGGTKGTCTACCC 3' | amplification | F^1,^ * |
|  | HBB-T1 | HBB_T1_RB_R19 | 5' ACAGGCAAGTGCAGGAAAG 3' | amplification | R^2,^ * |
|  | HBB-T1, HBB-T2 | HBB_T1_RB_FS16 | 5' GACCTGTCCTCTGCCT 3' | sequencing | F |
|  | HBB-T2 | HBB_T2_RB_F21 | 5' TTGGACCCAGAGGTTCTTTGA 3' | amplification | F ˜ |
|  | HBB-T2 | HBB_T2_RB_R21 | 5' CTCCAGGGGACAAAAAACATT 3' | amplification | R ˜ |
| Sanger sequencing | HBB-T1 | BT1F1 | 5' ACAYTTGCTTCTGACATAGT 3' | amplification, sequencing | F ^×^ |
|  | HBB-T1 | BT1R593 | 5' TGAAAGTAAATGCCTTTTATTAGT 3' | amplification, sequencing | R ^×^ |
|  | HBB-T2 | HBB10U19 | 5' ATGCACACCCTGGAATTGG 3' | amplification, sequencing | F ^+^ |
|  | HBB-T2 | HBB1266L21 | 5' GTGCATAAACACGAGCAAGAA 3' | amplification | R ^+^ |
|  | HBB-T2 | HBB597U19 | 5' CCCGTGGTTTCCTTCCTCT 3' | sequencing | F |

^1^ F – forward primer, ^2^ R – reverse primer; * expected amplicon length 937 bp; ˜ expected amplicon length 934 bp; ^×^ expected amplicon length 1282 bp; ^+^ expected amplicon length 1277 bp

Table S3. Bioclimatic variables used for Hb genotype–environment analysis as available at <http://www.worldclim.org/bioclim> and their abbreviations used in text.

| Variable ID | Variable | Abbreviation |
| --- | --- | --- |
| BIO1 | Annual Mean Temperature | AMT |
| BIO2 | Mean Diurnal Range | TDrange |
| BIO3 | Isothermality (BIO2/BIO7)o100 | Isotherm |
| BIO4 | Temperature Seasonality (st. dev. o 100) | Tseason |
| BIO5 | Max Temperature of Warmest Month | MaxTwarm |
| BIO6 | Min Temperature of Coldest Month | MinTcold |
| BIO7 | Temperature Annual Range (BIO5-BIO6) | TArange |
| BIO8 | Mean Temperature of Wettest Quarter | MeanTwetQ |
| BIO9 | Mean Temperature of Driest Quarter | MeanTdryQ |
| BIO10 | Mean Temperature of Warmest Quarter | MeanTwarmQ |
| BIO11 | Mean Temperature of Coldest Quarter | MeanTcoldQ |
| BIO12 | Annual Precipitation | AP |
| BIO13 | Precipitation of Wettest Month | Pwet |
| BIO14 | Precipitation of Driest Month | Pdry |
| BIO15 | Precipitation Seasonality (Coef. of variation) | Pseason |
| BIO16 | Precipitation of Wettest Quarter | PwetQ |
| BIO17 | Precipitation of Driest Quarter | PdryQ |
| BIO18 | Precipitation of Warmest Quarter | PwarmQ |
| BIO19 | Precipitation of Coldest Quarter | PcoldQ |

Table S4. List of samples selected for whole Hb gene sequencing by Sanger sequencing method.

| Sample | Locality | Country | Population | Latitude | Longitude | HBB-T1 genotype | HBB-T2 genotype | |
| --- | --- | --- | --- | --- | --- | --- | --- | --- |
| 18 | Feofania Forest | Ukraine | UA1 | 50.33 | 30.47 | Ser | Ser |  |
| 271 | Solnik | Bulgaria | BGR4 | 42.93 | 27.65 | Cys | Cys/Ser |  |
| 273 | Elena | Bulgaria | BGR4 | 42.88 | 25.86 | Cys/Ser | Ser |  |
| 274 | Bacau | Romania | RO2 | 46.55 | 26.83 | Ser | Ser |  |
| 367 | Frydek Mistek | Czech Republic | CZE4 | 49.70 | 18.38 | Cys/Ser | Ser |  |
| 368 | Frydek Mistek | Czech Republic | CZE4 | 49.70 | 18.38 | Ser | Ser |  |
| 369 | Frydek Mistek | Czech Republic | CZE4 | 49.70 | 18.38 | Cys/Ser | Ser |  |
| 370 | Frydek Mistek | Czech Republic | CZE4 | 49.70 | 18.38 | Ser | Ser |  |
| 377 | Ostrava | Czech Republic | CZE4 | 49.80 | 18.22 | Ser | Ser |  |
| 378 | Ostrava | Czech Republic | CZE4 | 49.80 | 18.22 | Ser | Cys/Ser |  |
| 379 | Ostrava | Czech Republic | CZE4 | 49.80 | 18.22 | Ser | Ser |  |
| 380 | Ostrava | Czech Republic | CZE4 | 49.80 | 18.22 | Ser | Cys/Ser |  |
| 450 | Strmosten | Serbia | SRB1 | 44.09 | 21.64 | Cys/Ser | Cys/Ser |  |
| 601 | Bezau | Austria | CHEATIT | 47.37 | 9.97 | Cys | Ser |  |
| 622 | Msec | Czech Republic | CZE1 | 50.21 | 13.90 | Cys | Cys/Ser |  |
| 623 | Msec | Czech Republic | CZE1 | 50.21 | 13.90 | Cys/Ser | Ser |  |
| 649 | Bursa, Uludag Mts | Turkey | TUR1 | 40.12 | 29.17 | Ser | Ser |  |
| 707 | Hakel forest | Germany | DE4 | 51.78 | 11.15 | Cys | Cys |  |
| 734 | Polichno | Poland | PL2 | 51.47 | 19.82 | Cys | Cys/Ser |  |
| 735 | Polichno | Poland | PL2 | 51.47 | 19.82 | Cys/Ser | Ser |  |
| 737 | Bialowieza National Park | Poland | PL4 | 52.73 | 23.85 | Ser | Ser |  |
| 755 | Ostashkhov | Russia | RUS1 | 57.15 | 33.10 | Ser | Ser |  |
| 836 | Beauvoir | France | FRA2 | 48.66 | -1.50 | Cys | Cys |  |
| 1048 | Geneva | Switzerland | CHE1 | 46.20 | 6.17 | Ser | N/A |  |
| 1126 | Bussieres | France | FRA6 | 46.04 | 2.65 | Cys | Cys/Ser |  |
| 1187 | Hanisberg | Slovakia | SK1 | 48.40 | 19.09 | Cys | Cys/Ser |  |
| 1188 | Hanisberg | Slovakia | SK1 | 48.40 | 19.09 | Cys/Ser | Ser |  |
| 1327 | Ratzerburg | Germany | DE3 | 53.68 | 10.75 | Cys | Cys/Ser |  |
| 1395 | Ribeaville | France | FRA4 | 50.17 | 1.63 | Ser | N/A |  |
| 1433 | Pyrenees | France | FRA7 | 43.25 | -0.12 | Ser | Ser |  |
| 1435 | Pyrenees | France | FRA7 | 43.25 | -0.12 | Cys/Ser | Ser |  |
| 1517 | Noordwijk | Netherlands | NLD1 | 52.25 | 4.47 | Cys | Cys/Ser |  |
| 1641 | Skog | Sweden | SWE3 | 61.14 | 16.81 | Cys/Ser | Ser |  |
| 1642 | Skog | Sweden | SWE3 | 61.14 | 16.81 | Cys/Ser | Ser |  |
| 1643 | Skog | Sweden | SWE3 | 61.14 | 16.81 | Cys/Ser | Ser |  |
| 1650 | Bollnas | Sweden | SWE3 | 61.45 | 16.28 | Cys/Ser | Ser |  |
| 1651 | Bollnas | Sweden | SWE3 | 61.46 | 16.28 | Cys/Ser | Ser |  |
| 1652 | Bollnas | Sweden | SWE3 | 61.46 | 16.28 | Cys/Ser | Ser |  |
| 1670 | Kramfors | Sweden | SWE1 | 62.88 | 17.75 | Ser | Ser |  |
| 1676 | Buckarby | Sweden | SWE3.2 | 60.22 | 16.98 | Cys/Ser | Ser |  |
| 1677 | Buckarby | Sweden | SWE3.2 | 60.22 | 16.98 | Cys/Ser | Cys/Ser |  |
| Sample | Locality | Country | Population | Latitude | Longitude | HBB-T1 allele | HBB-T2 allele | |
| 1692 | Harbo | Sweden | SWE3.2 | 60.09 | 17.32 | Ser | Ser |  |
| 1698 | Arla | Sweden | SWE5 | 59.29 | 16.70 | Cys | Cys/Ser |  |
| 1700 | Arla | Sweden | SWE5 | 59.30 | 16.79 | Cys | Ser |  |
| 1701 | Arla | Sweden | SWE5 | 59.30 | 16.79 | Ser | Ser |  |
| 1706 | Valla | Sweden | SWE5 | 59.00 | 16.36 | Cys/Ser | Ser |  |
| 1707 | Valla | Sweden | SWE5 | 59.00 | 16.36 | Cys | Ser |  |
| 1708 | Valla | Sweden | SWE5 | 59.00 | 16.36 | Ser | Ser |  |
| 1718 | Stensoffa, Kalvsmosse North | Sweden | SWE6 | 55.71 | 13.49 | Ser | Ser |  |
| 1719 | Stensoffa, Kalvsmosse North | Sweden | SWE6 | 55.71 | 13.49 | Ser | Ser |  |
| 1721 | Stensoffa, Ekskogen | Sweden | SWE6 | 55.69 | 13.46 | Ser | Ser |  |
| 1741 | Torsby | Sweden | SWE2 | 60.19 | 12.74 | Ser | Ser |  |
| 1742 | Torsby | Sweden | SWE2 | 60.19 | 12.74 | Cys/Ser | Cys/Ser |  |
| 1744 | Torsby | Sweden | SWE2 | 60.17 | 12.76 | Cys/Ser | Ser |  |
| 1765 | Strandkaer | Denmark | DNK1 | 56.23 | 10.57 | Ser | Ser |  |
| 1826 | Calabria, Catena Costiera | Italy | IT3 | 39.30 | 16.12 | Ser | Ser |  |
| 1828 | Calabria, Sila Grande | Italy | IT3 | 39.35 | 16.49 | Ser | Cys/Ser |  |
| 1845 | Apulia | Italy | IT2 | 41.82 | 16.01 | Ser | Ser |  |
| 1848 | Snasa | Norway | NOR2 | 64.17 | 12.05 | Ser | Ser |  |
| 1859 | Venabygd | Norway | NOR5 | 61.58 | 10.06 | Ser | Ser |  |
| 1867 | Eksingedalen | Norway | NOR4 | 60.79 | 5.97 | Ser | Ser |  |
| 1871 | Eksingedalen | Norway | NOR4 | 60.79 | 5.97 | Ser | Ser |  |
| 1879 | Lillesand | Norway | NOR6 | 58.24 | 8.23 | Ser | Ser |  |
| 1901 | Goteborg | Sweden | SWE4 | 57.62 | 12.42 | Ser | Ser |  |
| 1903 | Viterbo | Italy | IT1 | 42.52 | 12.12 | Ser | Ser |  |
| 1911 | Armendarits | France | FRA7 | 43.30 | 1.17 | Ser | Ser |  |

Table S5. Allelic cytonuclear disequilibria for Europe (EU) and for the combination Europe and Britain (EU+GB). Results show the non-random association between β52Cys alleles at the nuclear locus and three major mtDNA lineages. Statistical significance (α = 0.05) was assessed using the asymptotic test. Normalised estimates take into account the bounds imposed by marginal frequencies of the two markers.

|  | HBB-T1 52Cys | | | HBB-T2 52Cys | | |
| --- | --- | --- | --- | --- | --- | --- |
| Disequilibria | Western | Eastern | Carpathian | Western | Eastern | Carpathian |
| EU estimate | 0.084 | -0.032 | -0.037 | 0.075 | -0.021 | -0.039 |
| Normalised estimate | 0.381 | -0.558 | -0.256 | 0.667 | -0.846 | -0.615 |
| EU + GB estimate | 0.074 | -0.031 | -0.025 | 0.054 | -0.014 | -0.031 |
| Normalised estimate | 0.349 | -0.624 | -0.134 | 0.559 | -0.830 | -0.484 |

Table S6. Loadings of variables comprising principal components for the datasets containing data for continental Europe (EU), Britain (GB) and for the combined dataset (EU+GB). Loadings > |0.5| are highlighted.

|  | EU | | | | GB | | EU + GB | | | |
| --- | --- | --- | --- | --- | --- | --- | --- | --- | --- | --- |
| Variable | PC1 | PC2 | PC3 | PC4 | PC1 | PC2 | PC1 | PC2 | PC3 | PC4 |
| AMT | 0.470 | -0.808 | 0.216 | 0.229 | 0.141 | 0.968 | 0.202 | -0.874 | 0.367 | 0.187 |
| TDrange | 0.514 | 0.078 | 0.750 | -0.210 | 0.931 | -0.328 | 0.604 | 0.088 | 0.584 | -0.377 |
| Isotherm | 0.107 | -0.630 | 0.434 | -0.365 | 0.431 | -0.795 | -0.180 | -0.680 | 0.198 | -0.445 |
| Tseason | 0.458 | 0.767 | 0.293 | 0.141 | 0.906 | 0.206 | 0.657 | 0.657 | 0.267 | 0.072 |
| MaxTwarm | 0.741 | -0.335 | 0.514 | 0.224 | 0.839 | 0.487 | 0.709 | -0.315 | 0.610 | 0.101 |
| MinTcold | 0.056 | -0.964 | -0.095 | 0.141 | -0.550 | 0.790 | -0.292 | -0.925 | 0.016 | 0.144 |
| TArange | 0.521 | 0.651 | 0.488 | 0.041 | 0.956 | -0.073 | 0.697 | 0.569 | 0.376 | -0.055 |
| MeanTwetQ | 0.703 | 0.235 | 0.164 | 0.088 | 0.621 | 0.070 | 0.753 | 0.121 | 0.189 | 0.044 |
| MeanTdryQ | -0.218 | -0.768 | -0.083 | 0.205 | -0.729 | 0.490 | -0.447 | -0.671 | 0.059 | 0.241 |
| MeanTwarmQ | 0.719 | -0.458 | 0.348 | 0.314 | 0.504 | 0.816 | 0.634 | -0.466 | 0.531 | 0.249 |
| MeanTcoldQ | 0.140 | -0.968 | -0.006 | 0.125 | -0.508 | 0.840 | -0.220 | -0.949 | 0.086 | 0.112 |
| AP | -0.949 | -0.049 | 0.259 | 0.141 | -0.958 | -0.139 | -0.923 | 0.222 | 0.297 | 0.069 |
| Pwet | -0.893 | 0.095 | 0.251 | 0.329 | -0.977 | -0.042 | -0.837 | 0.349 | 0.315 | 0.243 |
| Pdry | -0.842 | -0.166 | 0.369 | -0.246 | -0.961 | -0.199 | -0.867 | 0.068 | 0.304 | -0.305 |
| Pseason | 0.041 | 0.582 | -0.074 | 0.666 | -0.932 | 0.248 | 0.109 | 0.572 | 0.061 | 0.668 |
| PwetQ | -0.900 | 0.088 | 0.245 | 0.328 | -0.976 | -0.059 | -0.851 | 0.334 | 0.306 | 0.240 |
| PdryQ | -0.868 | -0.202 | 0.350 | -0.177 | -0.936 | -0.251 | -0.889 | 0.057 | 0.318 | -0.250 |
| PwarmQ | -0.695 | 0.382 | 0.387 | -0.062 | -0.844 | -0.400 | -0.538 | 0.622 | 0.341 | -0.174 |
| PcoldQ | -0.900 | -0.217 | 0.115 | 0.211 | -0.983 | 0.016 | -0.933 | 0.016 | 0.182 | 0.181 |
| % explainedo | 41.4 | 29.0 | 11.4 | 6.9 | 65.3 | 24.0 | 42.9 | 29.1 | 10.8 | 7.0 |
| cumulative % |  | 70.5 | 81.9 | 88.7 |  | 89.3 |  | 72.0 | 82.8 | 89.8 |

o percentage of total variance explained by the component

Table S7. Spearman’s rho and associated *p*-value for correlation between HBB-T1 52Cys allele frequency and latitude, longitude, altitude and principal components identified in PCA. Results shown for the datasets of continental Europe (EU), Britain (GB) and for the combined dataset (EU+GB). *p* < 0.05 is underlined. For variables comprising the principal components and their loadings see Table 6.

|  | EU | | GB | | EU + GB | |
| --- | --- | --- | --- | --- | --- | --- |
| Component | rho | *p* | rho | *p* | rho | *p* |
| Latitude | -0.1042 | 0.3907 | -0.8810 | 0.0002 | -0.1374 | 0.2182 |
| Longitude | -0.3298 | 0.0053 | 0.2792 | 0.3795 | -0.3324 | 0.0023 |
| Altitude | -0.2449 | 0.0410 | 0.1414 | 0.6611 | -0.2637 | 0.0167 |
| PC1 | 0.2074 | 0.0850 | 0.3444 | 0.2729 | 0.0754 | 0.5009 |
| PC2 | -0.3996 | 0.0006 | 0.8194 | 0.0011 | -0.5050 | 0.000001 |
| PC3 | -0.0040 | 0.9735 |  |  | -0.0528 | 0.6377 |
| PC4 | -0.3661 | 0.0018 |  |  | -0.2485 | 0.0243 |

Table S8. Result from Samβada analysis for continental Europe dataset, 1 variable models. Only significant models with significant parents are shown (according to Wald score). Bonferroni adjusted *p*‑value associated with Wald score is *p* = 0.00044.

Table S9. Result from Samβada analysis for continental Europe dataset, 2 variable models. Only significant models with significant parents are shown (according to Wald score). Bonferroni adjusted *p*‑value associated with Wald score is *p* = 0.000039.

Table S10. Result from Samβada analysis for continental Europe dataset, 3 variable models. Only significant models with significant parents are shown (according to Wald score). Bonferroni adjusted *p*‑value associated with Wald score is *p* = 0.0000057.

Table S11. Result from Samβada analysis for continental Europe dataset, 4 variable models. Only significant models with significant parents are shown (according to Wald score). Bonferroni adjusted *p*‑value associated with Wald score is *p* = 0.0000011.

Table S12. Result from Samβada analysis for Britain dataset, 1 variable models. Only significant models with significant parents are shown (according to Wald score). Bonferroni adjusted *p*‑value associated with Wald score is *p* = 0.00044.

Table S13. Result from Samβada analysis for Britain dataset, 2 variable models. Only significant models with significant parents are shown (according to Wald score). Bonferroni adjusted *p*‑value associated with Wald score is *p* = 0.000039.

Table S14. Result from Samβada analysis for Britain dataset, 3 variable models. Only significant models with significant parents are shown (according to Wald score). Bonferroni adjusted *p*‑value associated with Wald score is *p* = 0.0000057.

Table S15. Result from Samβada analysis for combined dataset (Britain + continental Europe), 1 variable models. Only significant models with significant parents are shown (according to Wald score). Bonferroni adjusted *p*‑value associated with Wald score is *p* = 0.00044.

Table S16. Result from Samβada analysis for combined dataset (Britain + continental Europe), 2 variable models. Only significant models with significant parents are shown (according to Wald score). Bonferroni adjusted *p*‑value associated with Wald score is *p* = 0.000039.

Table S17. Result from Samβada analysis for combined dataset (Britain + continental Europe), 3 variable models. Only significant models with significant parents are shown (according to Wald score). Bonferroni adjusted *p*‑value associated with Wald score is *p* = 0.0000057.

Table S18. Result from Samβada analysis for combined dataset (Britain + continental Europe), 4 variable models. Only significant models with significant parents are shown (according to Wald score). Bonferroni adjusted *p*‑value associated with Wald score is *p* = 0.0000011.

Table S19. Result from Samβada analysis for Western and Carpathian clades (as defined by mtDNA phylogeography), 1, 2 and 3 variables models. Only significant models with significant parents are shown (according to Wald score). Bonferroni adjusted *p*‑value associated with Wald score is *p* = 0.00044, *p* = 0.000039, *p* = 0.0000057 respectively.

Due to large size, the tables are provided in separate Excel file Tables S8-S19_Supporting_information.xlsx, each table on separate sheet.

Table S20. Alignment of HBB-T1 and HBB-T2 haplotypes, showing variable sites only. Identified gene conversion tracts are shown. HBB-T1 haplotypes are in grey, HBB-T2 haplotypes in white. Coding regions of the beta globin gene are marked by black boxes. Ser52Cys position is marked by orange column, position determining complete/incomplete Chi sequence is marked by red box. Conversion tracts identified by method of Betrán *et al.* (2007) are in grey/white depending on their gene of origin. Conversion tracts identified by GENECONV are in yellow.

Due to the large size of this table, it is provided as a separate Excel file, Table S20_Supporting_information.xlsx.


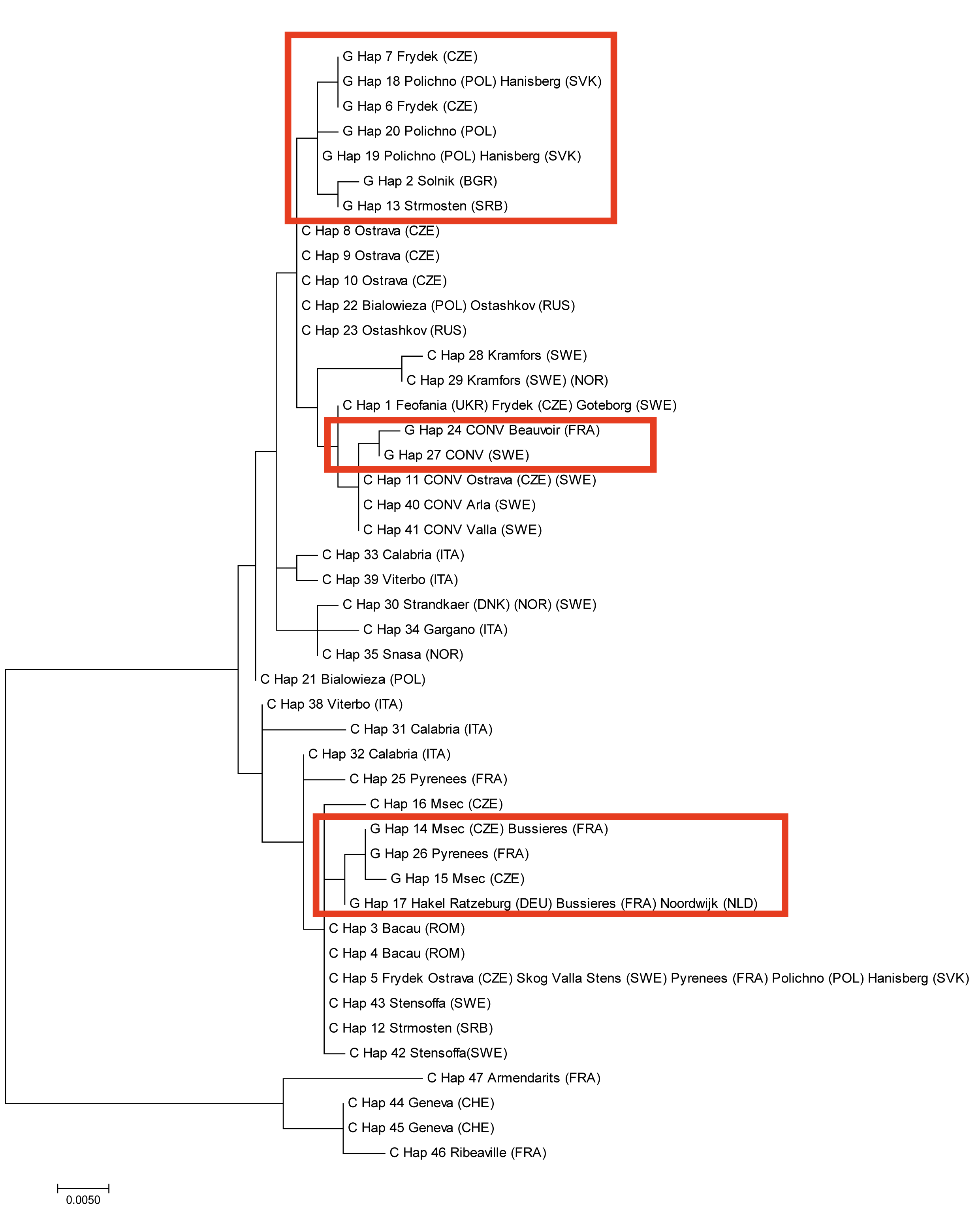


Figure S1. Maximum likelihood phylogeny of HBB-T1 haplotypes based on the alignment segment left of the breakpoint at site 521. C and G letters represent the Ser and Cys allele, respectively. Haplotypes containing the Cys allele are boxed.


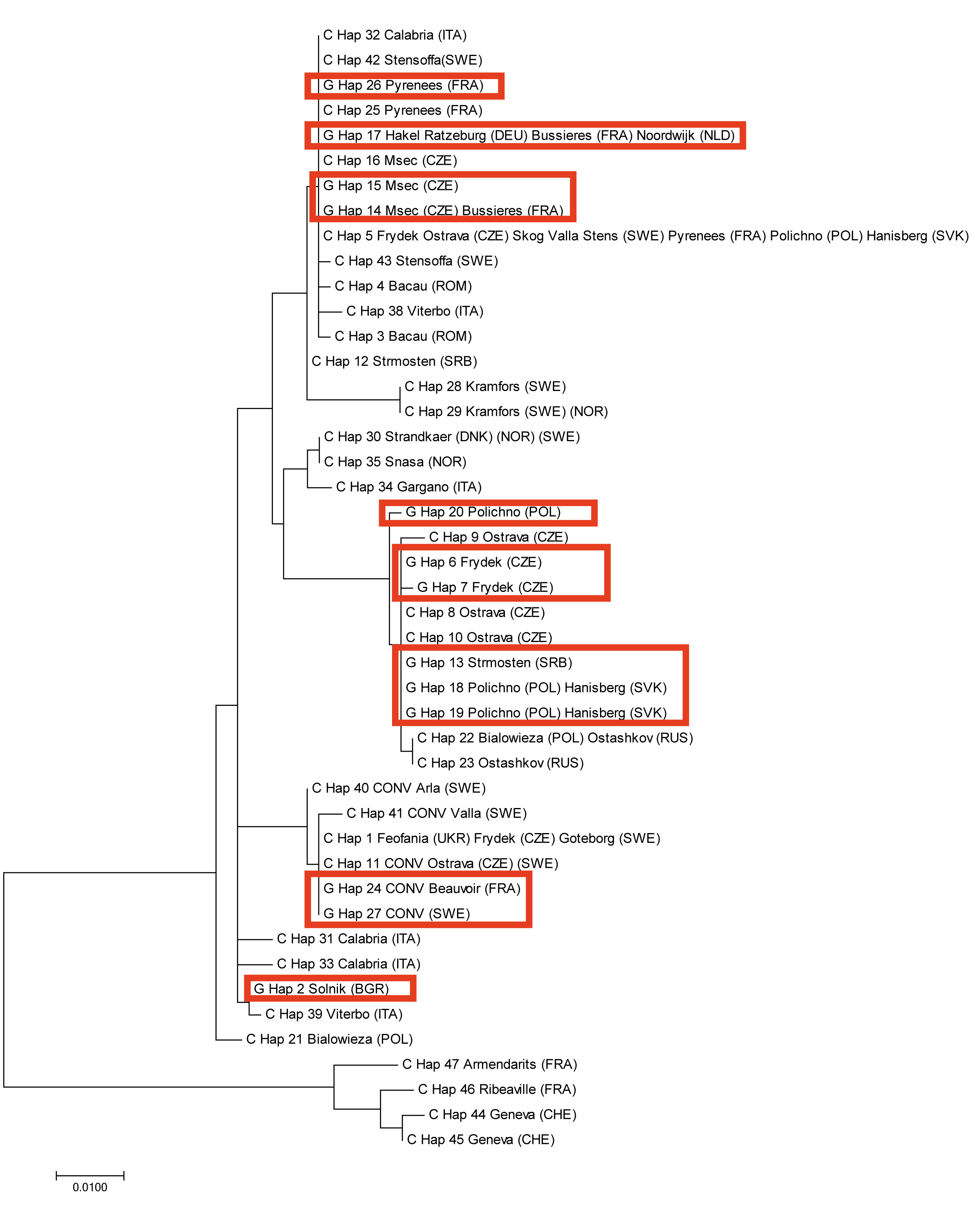


Figure S2. Maximum likelihood phylogeny of HBB-T1 haplotypes based on the alignment segment right of the breakpoint at site 521. C and G letters represent the Ser and Cys allele, respectively. Haplotypes containing the Cys allele are boxed.


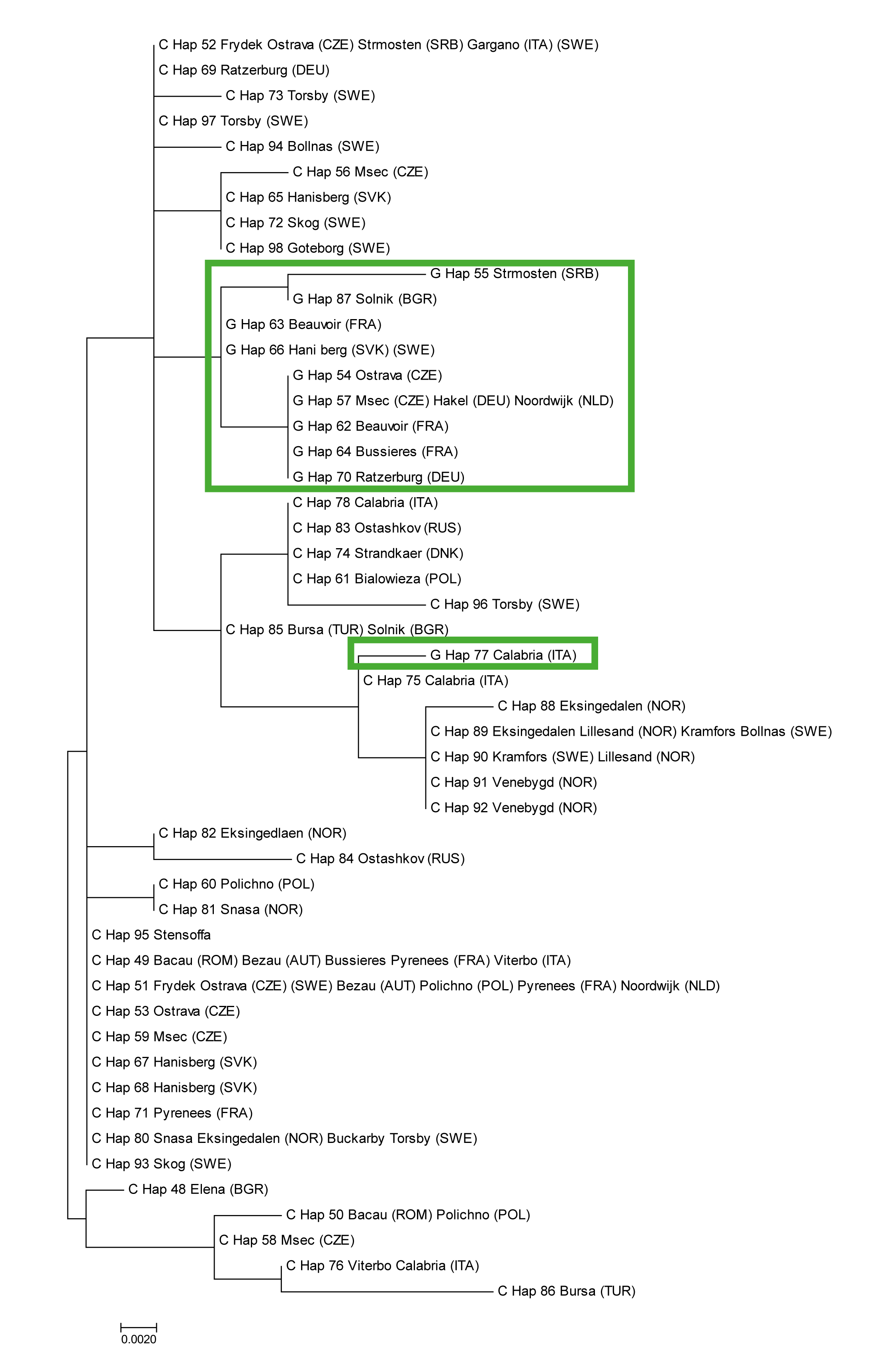


Figure S3. Maximum likelihood phylogeny of HBB-T2 haplotypes based on the alignment segment left of the breakpoint at site 329. C and G letters represent the Ser and Cys allele, respectively. Haplotypes containing the Cys allele are boxed.


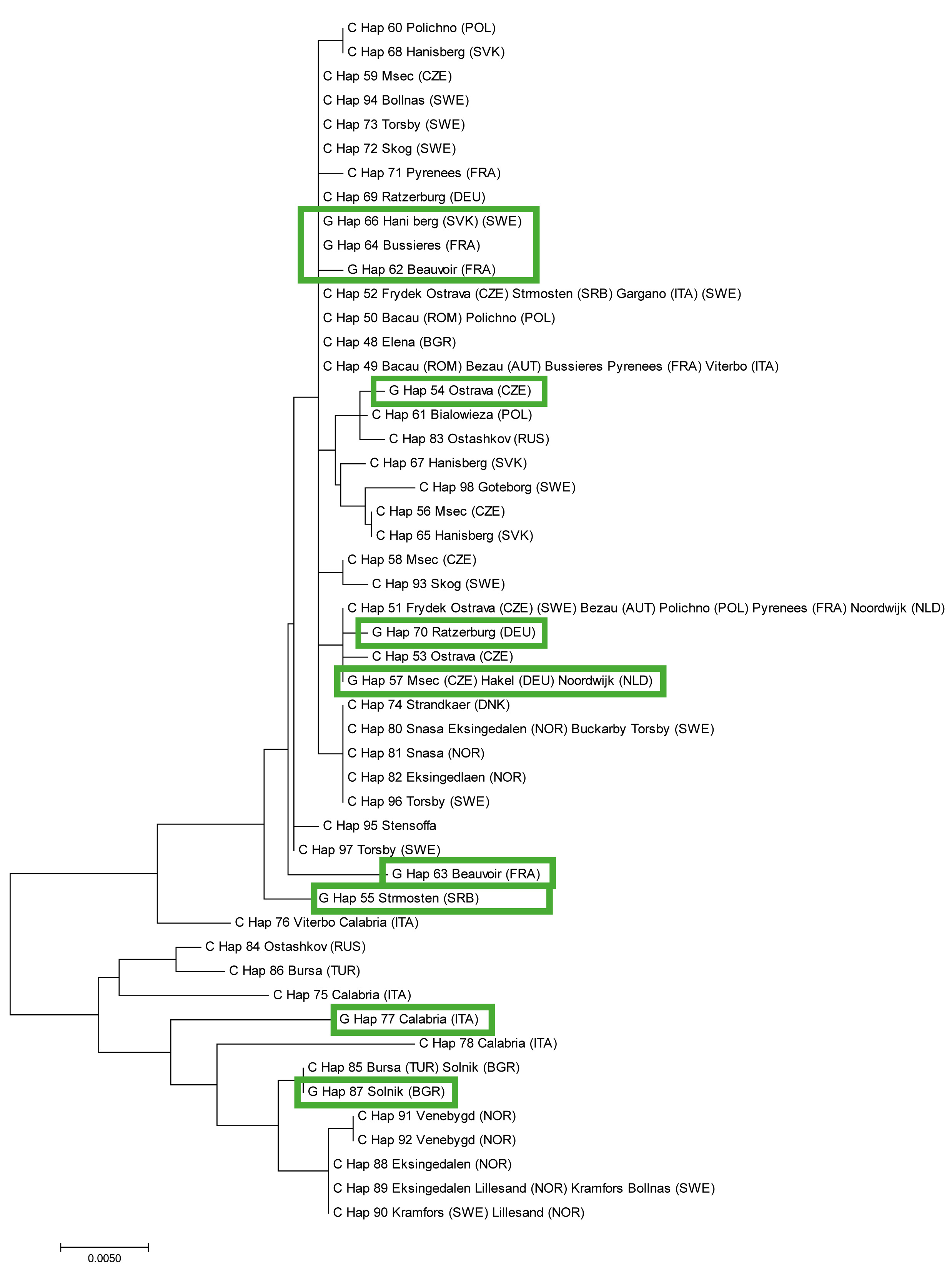


Figure S4. Maximum likelihood phylogeny of HBB-T2 haplotypes based on the alignment segment right of the breakpoint at site 329. C and G letters represent the Ser and Cys allele, respectively. Haplotypes containing the Cys allele are boxed.


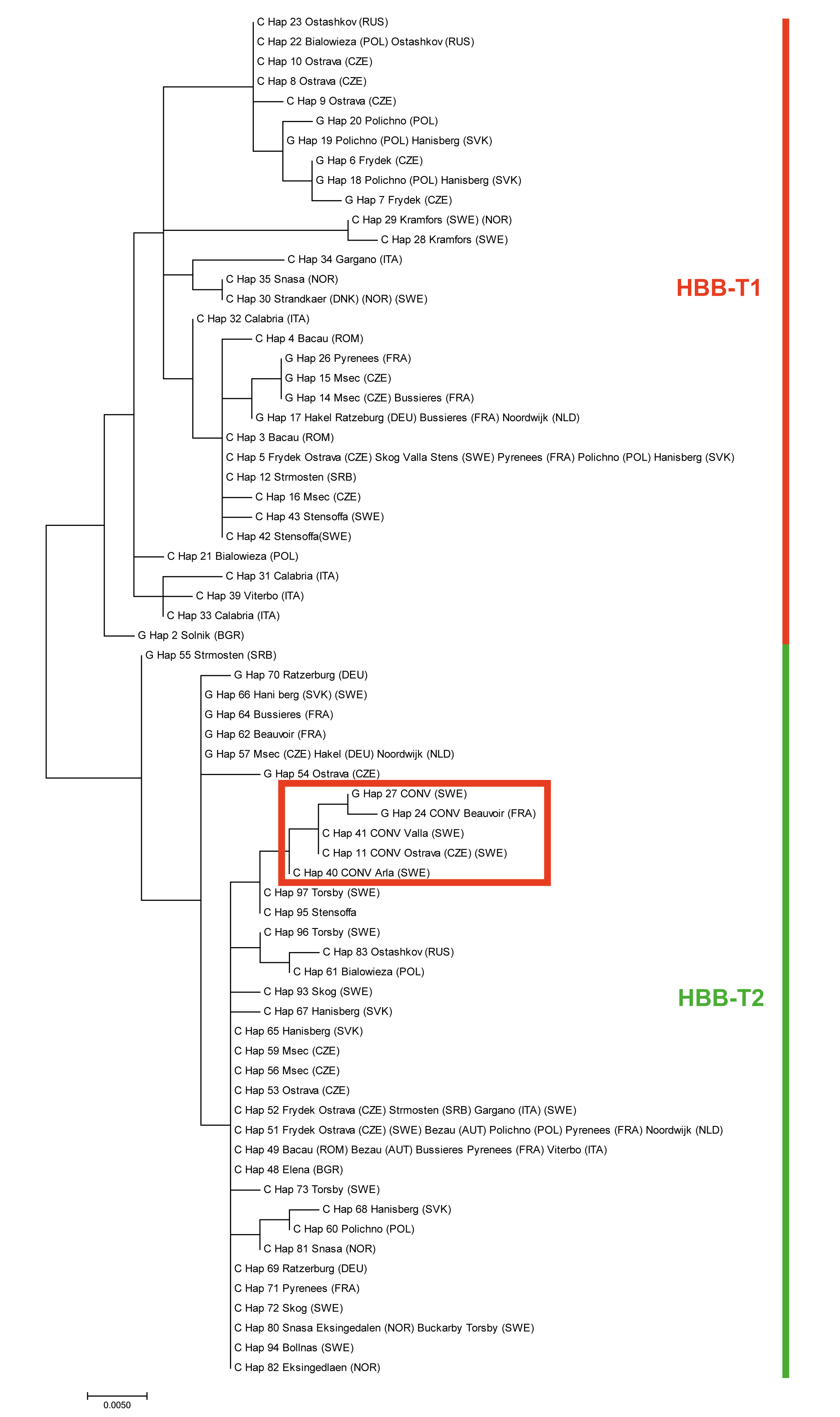


Figure S5. Maximum likelihood phylogeny for both genes representing the converted gene segment spanning sites 208 - 664. Other haplotypes containing conversion tracts are excluded. C and G letters represent the Ser and Cys allele, respectively. Converted haplotypes are boxed.


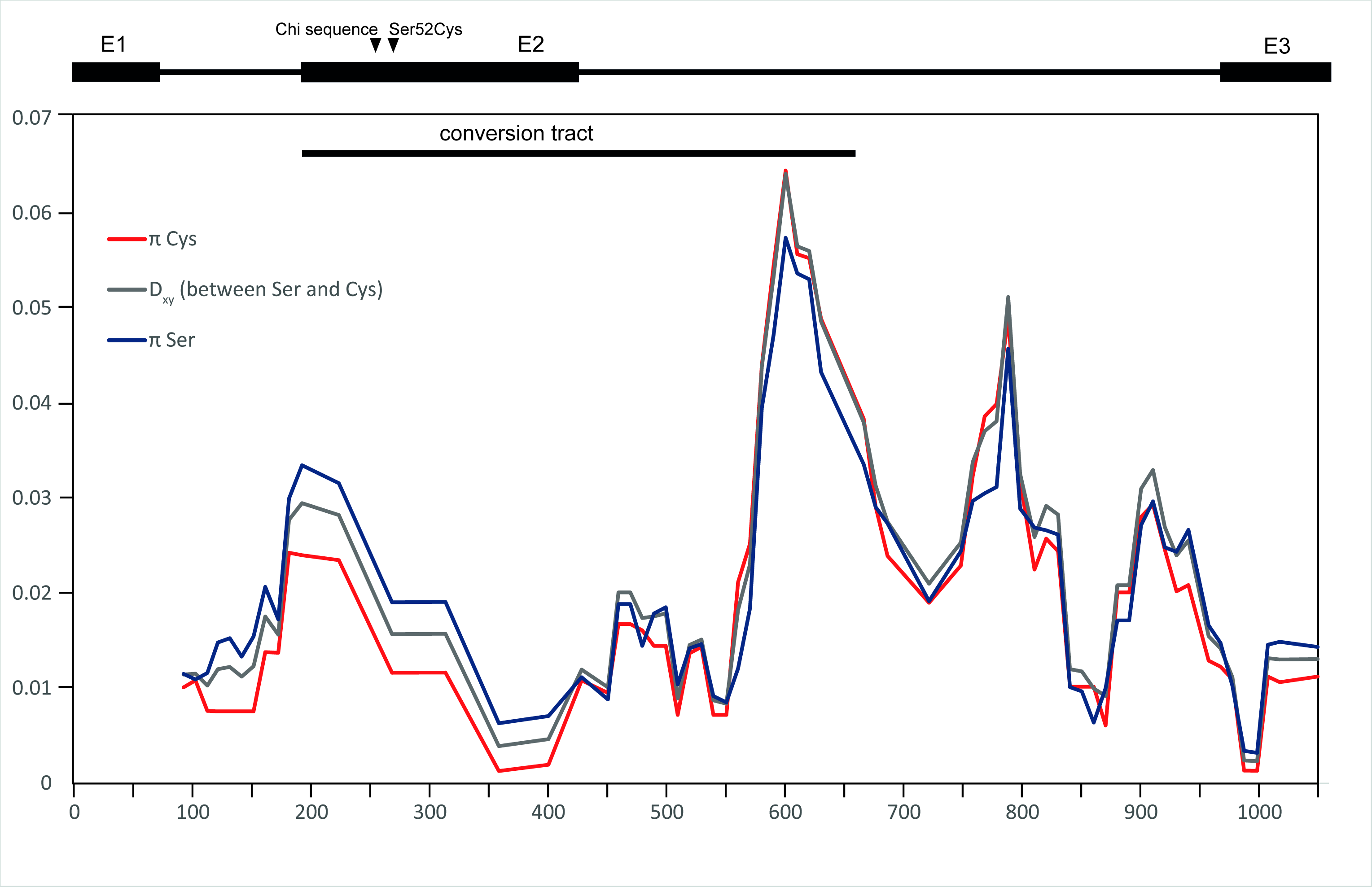


Figure S6. Results of a sliding window test of silent site diversity within (π) and between (D_xy_) the Cys and Ser haplotypes. Exons (E1-3) are marked by black rectangles (top).
