# Supplementary material for: Playing Hide-and-Seek in Beta-Globin Genes: Gene Conversion Transferring a Beneficial Mutation between Differentially Expressed Gene Duplicates
Source: Genes (Basel). 2018 Oct 12;9(10):492. doi: 10.3390/genes9100492 (PMC6209878; doi:10.3390/genes9100492)
Supplement: Supplementary file 1 [file genes-09-00492-s001.zip › Supporting_information.pdf]

Supporting information:

Table S1. List of the 72 population samples derived from the 136 sampling localities.

Population	Population latitude	Population longitude	Nr. of samples in population	HBB-T1 Cys frequency	HBB-T2 Cys frequency	Country	Locality latitude	Locality longitude	Locality altitude (m)	Nr. of samples
AT1	47.85	15.05	10	0.000	0.000	Austria	47.85	15.05	880*	10
BEL1	50.63	4.18	5	1.000	1.000	Belgium	50.63	4.18	99	5
BGR1	43.10	23.40	4	0.250	0.125	Bulgaria	43.10	23.40	618	4
BGR2	42.20	23.00	5	0.600	0.100	Bulgaria	42.20	23.00	766	5
BGR3	41.50	24.50	3	0.833	0.167	Bulgaria	41.50	24.50	1267	3
BGR4	42.91	27.05	3	0.833	0.333	Bulgaria	42.93	27.65	197*	2
						Bulgaria	42.88	25.86	618*	1
CZAT	48.84	16.66	11	0.818	0.000	Austria	48.37	16.23	170*	1
						Czech Republic	48.97	16.60	166*	5
						Czech Republic	48.80	16.80	157*	5
CZE1	50.23	13.95	11	0.909	0.591	Czech Republic	50.21	13.90	378*	10
						Czech Republic	50.42	14.47	260	1
CZE2	49.57	14.90	12	0.417	0.125	Czech Republic	49.80	18.22	548	11
						Czech Republic	48.98	14.43	475*	1
CZE3	50.50	16.12	6	0.417	0.000	Czech Republic	50.50	16.12	500	6
CZE4	49.75	18.30	10	0.100	0.100	Czech Republic	49.70	18.38	318*	5
						Czech Republic	49.80	18.22	201*	5
CZSK	48.86	18.58	11	0.545	0.000	Czech Republic	49.01	17.76	287*	1
						Slovakia	48.84	18.66	282*	10
DE1	53.62	7.27	10	1.000	0.200	Germany	53.62	7.27	-1	10
DE2	54.22	9.11	10	0.150	0.000	Germany	54.22	9.11	26	8
						Germany	54.23	9.11	1	2
DE3	53.65	10.78	12	0.917	0.417	Germany	53.68	10.75	30	10
						Germany	54.21	11.07	2	1
						Germany	52.80	10.83	86	1
DE4	51.85	11.18	10	1.000	0.950	Germany	51.78	11.15	122	9
						Germany	52.50	11.46	73	1
DE5	51.86	13.06	10	1.000	1.000	Germany	51.86	13.06	96	10
DE6	50.71	11.87	3	0.667	0.500	Germany	50.17	11.38	470	1
						Germany	50.97	12.14	293	1
						Germany	50.99	12.08	293	1
DE7	49.02	10.73	7	1.000	0.143	Germany	49.02	10.73	542	7
DNK1	55.80	9.41	5	0.000	0.000	Denmark	55.63	8.48	19	2
						Denmark	55.27	8.94	19	1
						Denmark	56.23	10.57	57	2
DNK2	54.96	11.63	7	0.000	0.000	Denmark	55.73	11.92	17	1
						Denmark	54.87	11.71	5	2
						Denmark	54.83	11.67	4	2
						Denmark	54.81	11.36	8	2
FRA1	47.34	-3.17	1	-	1.000	France	47.34	-3.17		1
FRA2	48.60	-1.50	4	1.000	1.000	France	48.60	-1.50	15	4

Population	Population latitude	Population longitude	Nr. of samples in population	HBB-T1 Cys frequency	HBB-T2 Cys frequency	Country	Locality latitude	Locality longitude	Locality altitude (m)	Nr. of samples
FRA3	48.97	0.13	5	1.000	1.000	France	48.92	0.20	99	4
						France	49.17	-0.15	1	1
FRA4	50.53	1.78	9	0.889	0.938	France	50.08	1.57	71	1
						France	50.17	1.63	27	3
						France	50.87	1.82	26	3
						France	50.75	2.25	14	1
						France	50.83	1.87	109	1
FRA5	48.71	1.76	8	1.000	1.000	France	48.55	3.40	158	1
						France	48.75	1.55	136	2
						France	48.72	1.52	134	5
FRA6	46.04	2.60	11	0.955	0.455	France	45.70	2.13	823	1
						France	46.04	2.65	658	4
						France	46.14	2.73	598	2
						France	46.00	2.55	569	2
						France	46.17	2.63	481	2
FRA7	43.25	-0.12	10	0.650	0.000	France	43.25	-0.12	402	10
FRA7.2	43.30	1.17	4	0.750	0.250	France	43.30	1.17	221	4
HR1	45.74	15.65	6	0.000	0.000	Croatia	45.74	15.65	443*	6
HR2	44.84	15.62	10	0.000	0.000	Croatia	44.83	15.62	643*	2
						Croatia	44.85	15.62	570*	1
						Croatia	44.84	15.62	770	7
HU1	47.30	19.22	7	0.000	0.000	Hungary	47.30	19.22	102*	7
CHE1	46.38	6.35	9	0.056	0.000	Switzerland	46.20	6.17	425	4
						Switzerland	46.52	6.50	400	5
CHE2	45.90	7.20	6	0.000	0.000	Switzerland	45.90	7.20	2087	6
CHE3	47.37	8.55	2	0.750	0.000	Switzerland	47.37	8.55	424	2
CHEATIT	46.78	10.27	13	0.077	0.000	Switzerland	46.78	10.18	1450*	10
						Italy	46.07	11.11	193	1
						Austria	47.37	9.97	745*	1
						Austria	46.92	10.60	1450*	1
IRL1	53.11	-8.60	2	1.000	0.500	Ireland	53.08	-8.23	47	1
						Ireland	53.13	-8.97	14	1
IT1	42.87	11.59	6	0.000	0.000	Italy	42.52	12.12	329	3
						Italy	43.22	11.07	438	3
IT2	41.82	16.01	4	0.000	0.000	Italy	41.82	16.01	766	4
IT3	39.35	16.45	10	0.000	0.050	Italy	39.30	16.12	1021	1
						Italy	39.35	16.49	1176	9
NLD1	52.50	4.57	11	0.955	0.773	Netherlands	52.80	4.70	-4*	5
						Netherlands	52.25	4.47	-21*	6
NLD2	53.07	6.24	5	1.000	0.100	Netherlands	53.07	6.24	27*	5
NLD3	52.07	6.08	5	1.000	0.200	Netherlands	52.07	6.08	79*	5
NOR1	66.42	14.77	1	0.000	0.000	Norway	66.42	14.77	154*	1
NOR2	64.15	11.97	6	0.000	0.000	Norway	64.17	12.05	56*	2
						Norway	64.14	11.93	46*	4
NOR3	62.96	8.71	1	0.000	0.000	Norway	62.96	8.71	197*	1

Population	Population latitude	Population longitude	Nr. of samples in population	HBB-T1 Cys frequency	HBB-T2 Cys frequency	Country	Locality latitude	Locality longitude	Locality altitude (m)	Nr. of samples
NOR4	60.79	5.97	8	0.000	0.000	Norway	60.79	5.97	287*	8
NOR5	61.58	10.06	9	0.000	0.000	Norway	61.58	10.06	627*	8
						Norway	60.22	10.73		1
NOR6	58.31	8.18	12	0.000	0.000	Norway	58.98	7.66	226*	1
						Norway	58.24	8.23	115*	10
						Norway	58.77	9.21		1
PL1	52.45	17.10	4	0.500	0.375	Poland	52.45	17.10	272*	4
PL2	51.47	19.82	4	0.750	0.500	Poland	51.47	19.82	192*	4
PL3	53.80	21.65	10	0.000	0.000	Poland	53.80	21.65	145*	10
PL4	52.73	23.85	10	0.000	0.000	Poland	52.73	23.85	168	10
RO1	46.48	23.66	6	0.333	0.000	Romania	45.30	23.83	1722	1
						Romania	46.72	23.62	740	5
RO2	46.55	26.83	6	0.000	0.000	Romania	46.55	26.83	284*	6
RUS1	57.15	33.10	5	0.000	0.000	Russia	57.15	33.10	203	5
SK1	48.38	19.08	11	0.682	0.045	Slovakia	48.26	18.99	203*	1
						Slovakia	48.40	19.09	350*	10
SK2	49.16	20.07	11	0.273	0.000	Slovakia	49.20	19.75	1900	1
						Slovakia	49.13	20.16	1114*	1
						Slovakia	49.15	20.16	1491*	4
						Slovakia	49.17	19.98	1189*	2
						Slovakia	49.16	20.08	1531*	3
SK3	48.77	21.39	9	0.125	0.000	Slovakia	48.84	22.18	270*	1
						Slovakia	48.77	21.37	279	7
						Slovakia	48.70	20.69	611*	1
SRB1	44.09	21.64	4	0.625	0.125	Serbia	44.09	21.64	333*	4
SRB2	43.24	20.82	5	0.400	0.000	Serbia	43.24	20.82	1148	5
SRBHR	45.16571	19.58	7	0.571	0.000	Croatia	45.14	17.60	128*	1
						Serbia	45.17	19.91	172*	6
SVN1	46.11	14.67	6	0.000	0.000	Slovenia	46.11	14.67	227*	6
SWE1	62.88	17.74	10	0.000	0.000	Sweden	62.88	17.72	209	5
						Sweden	62.88	17.75	94	5
SWE2	60.17	12.77	10	0.200	0.050	Sweden	60.14	12.81	159*	3
						Sweden	60.17	12.76	188*	3
						Sweden	60.19	12.74	300*	3
						Sweden	60.17	12.76	208*	1
SWE3	61.30	16.55	10	0.400	0.000	Sweden	61.15	16.81	102	5
						Sweden	61.46	16.28	188	4
						Sweden	61.45	16.29	172	1
SWE3.2	60.18	17.09	3	0.333	0.167	Sweden	60.21	16.98	79	2
						Sweden	60.09	17.32	55	1
SWE4	57.62	12.42	1	0.000	0.000	Sweden	57.62	12.42	109*	1
SWE5	59.21	16.63	10	0.700	0.050	Sweden	59.29	16.70	54*	3
						Sweden	59.30	16.79	70*	4
						Sweden	59.00	16.36	72*	3

SWE6	55.70	13.47	10	0.000	0.000	Sweden	55.71	13.49	3*	5
						Sweden	55.69	13.46	37*	5
TUR1	40.12	29.17	10	0.000	0.000	Turkey	40.12	29.17	2002	10
UA1	50.41	29.72	6	0.000	0.000	Ukraine	50.33	30.47	147	5
							50.80	25.95	207	1

*Altitude data obtained by extraction from World Clim dataset at a 30 seconds resolution using ArcGIS v 10.2 (ESRI) Spatial analyst toolbox; altitude data of other localities captured in the field using a GPS (global positioning system) device.

Table S2. Primers used for pyrosequencing and Sanger sequencing. The sequencing primer designed for HBB-T1 pyrosequencing assays was used also for HBB-T2. Sanger sequencing primers were designed by Kotlík *et al.* (2014).

sequencing method	gene	primer	sequence	type	direction
pyrosequencing	HBB-T1	HBB_T1_RB_F19	5' GGCTGCTGGTKGTCTACCC 3'	amplification	F[1, *]
	HBB-T1	HBB_T1_RB_R19	5' ACAGGCAAGTGCAGGAAAG 3'	amplification	R[2, *]
	HBB-T1, HBB-T2	HBB_T1_RB_FS16	5' GACCTGTCCTCTGCCT 3'	sequencing	F
	HBB-T2	HBB_T2_RB_F21	5' TTGGACCCAGAGGTTCTTTGA 3'	amplification	F[~]
	HBB-T2	HBB_T2_RB_R21	5' CTCCAGGGGACAAAAAACATT 3'	amplification	R[~]
Sanger sequencing	HBB-T1	BT1F1	5' ACAYTTGCTTCTGACATAGT 3'	amplification, sequencing	F[×]
	HBB-T1	BT1R593	5' TGAAAGTAAATGCCTTTTATTAGT 3'	amplification, sequencing	R[×]
	HBB-T2	HBB10U19	5' ATGCACACCCTGGAATTGG 3'	amplification, sequencing	F[+]
	HBB-T2	HBB1266L21	5' GTGCATAAACACGAGCAAGAA 3'	amplification	R[+]
	HBB-T2	HBB597U19	5' CCCGTGGTTTCCTTCCTCT 3'	sequencing	F

[1] F – forward primer, [2] R – reverse primer; * expected amplicon length 937 bp; [~] expected amplicon length 934 bp; [×] expected amplicon length 1282 bp; [+] expected amplicon length 1277 bp

Table S3. Bioclimatic variables used for Hb genotype–environment analysis as available at http://www.worldclim.org/bioclim and their abbreviations used in text.

Variable ID	Variable	Abbreviation
BIO1	Annual Mean Temperature	AMT
BIO2	Mean Diurnal Range	TDrange
BIO3	Isothermality (BIO2/BIO7)o100	Isotherm
BIO4	Temperature Seasonality (st. dev. o 100)	Tseason
BIO5	Max Temperature of Warmest Month	MaxTwarm
BIO6	Min Temperature of Coldest Month	MinTcold
BIO7	Temperature Annual Range (BIO5-BIO6)	TArange
BIO8	Mean Temperature of Wettest Quarter	MeanTwetQ
BIO9	Mean Temperature of Driest Quarter	MeanTdryQ
BIO10	Mean Temperature of Warmest Quarter	MeanTwarmQ
BIO11	Mean Temperature of Coldest Quarter	MeanTcoldQ
BIO12	Annual Precipitation	AP
BIO13	Precipitation of Wettest Month	Pwet
BIO14	Precipitation of Driest Month	Pdry
BIO15	Precipitation Seasonality (Coef. of variation)	Pseason
BIO16	Precipitation of Wettest Quarter	PwetQ
BIO17	Precipitation of Driest Quarter	PdryQ
BIO18	Precipitation of Warmest Quarter	PwarmQ
BIO19	Precipitation of Coldest Quarter	PcoldQ

Table S4. List of samples selected for whole Hb gene sequencing by Sanger sequencing method.

Sample	Locality	Country	Population	Latitude	Longitude	HBB-T1 genotype	HBB-T2 genotype
18	Feofania Forest	Ukraine	UA1	50.33	30.47	Ser	Ser
271	Solnik	Bulgaria	BGR4	42.93	27.65	Cys	Cys/Ser
273	Elena	Bulgaria	BGR4	42.88	25.86	Cys/Ser	Ser
274	Bacau	Romania	RO2	46.55	26.83	Ser	Ser
367	Frydek Mistek	Czech Republic	CZE4	49.70	18.38	Cys/Ser	Ser
368	Frydek Mistek	Czech Republic	CZE4	49.70	18.38	Ser	Ser
369	Frydek Mistek	Czech Republic	CZE4	49.70	18.38	Cys/Ser	Ser
370	Frydek Mistek	Czech Republic	CZE4	49.70	18.38	Ser	Ser
377	Ostrava	Czech Republic	CZE4	49.80	18.22	Ser	Ser
378	Ostrava	Czech Republic	CZE4	49.80	18.22	Ser	Cys/Ser
379	Ostrava	Czech Republic	CZE4	49.80	18.22	Ser	Ser
380	Ostrava	Czech Republic	CZE4	49.80	18.22	Ser	Cys/Ser
450	Strmosten	Serbia	SRB1	44.09	21.64	Cys/Ser	Cys/Ser
601	Bezau	Austria	CHEATIT	47.37	9.97	Cys	Ser
622	Msec	Czech Republic	CZE1	50.21	13.90	Cys	Cys/Ser
623	Msec	Czech Republic	CZE1	50.21	13.90	Cys/Ser	Ser
649	Bursa, Uludag Mts	Turkey	TUR1	40.12	29.17	Ser	Ser
707	Hakel forest	Germany	DE4	51.78	11.15	Cys	Cys
734	Polichno	Poland	PL2	51.47	19.82	Cys	Cys/Ser
735	Polichno	Poland	PL2	51.47	19.82	Cys/Ser	Ser
737	Bialowieza National Park	Poland	PL4	52.73	23.85	Ser	Ser
755	Ostashkhov	Russia	RUS1	57.15	33.10	Ser	Ser
836	Beauvoir	France	FRA2	48.66	-1.50	Cys	Cys
1048	Geneva	Switzerland	CHE1	46.20	6.17	Ser	N/A
1126	Bussieres	France	FRA6	46.04	2.65	Cys	Cys/Ser
1187	Hanisberg	Slovakia	SK1	48.40	19.09	Cys	Cys/Ser
1188	Hanisberg	Slovakia	SK1	48.40	19.09	Cys/Ser	Ser
1327	Ratzerburg	Germany	DE3	53.68	10.75	Cys	Cys/Ser
1395	Ribeaville	France	FRA4	50.17	1.63	Ser	N/A
1433	Pyrenees	France	FRA7	43.25	-0.12	Ser	Ser
1435	Pyrenees	France	FRA7	43.25	-0.12	Cys/Ser	Ser
1517	Noordwijk	Netherlands	NLD1	52.25	4.47	Cys	Cys/Ser
1641	Skog	Sweden	SWE3	61.14	16.81	Cys/Ser	Ser
1642	Skog	Sweden	SWE3	61.14	16.81	Cys/Ser	Ser
1643	Skog	Sweden	SWE3	61.14	16.81	Cys/Ser	Ser
1650	Bollnas	Sweden	SWE3	61.45	16.28	Cys/Ser	Ser
1651	Bollnas	Sweden	SWE3	61.46	16.28	Cys/Ser	Ser
1652	Bollnas	Sweden	SWE3	61.46	16.28	Cys/Ser	Ser
1670	Kramfors	Sweden	SWE1	62.88	17.75	Ser	Ser
1676	Buckarby	Sweden	SWE3.2	60.22	16.98	Cys/Ser	Ser
1677	Buckarby	Sweden	SWE3.2	60.22	16.98	Cys/Ser	Cys/Ser

Sample	Locality	Country	Population	Latitude	Longitude	HBB-T1 allele	HBB-T2 allele
1692	Harbo	Sweden	SWE3.2	60.09	17.32	Ser	Ser
1698	Arla	Sweden	SWE5	59.29	16.70	Cys	Cys/Ser
1700	Arla	Sweden	SWE5	59.30	16.79	Cys	Ser
1701	Arla	Sweden	SWE5	59.30	16.79	Ser	Ser
1706	Valla	Sweden	SWE5	59.00	16.36	Cys/Ser	Ser
1707	Valla	Sweden	SWE5	59.00	16.36	Cys	Ser
1708	Valla	Sweden	SWE5	59.00	16.36	Ser	Ser
1718	Stensoffa, Kalvsmosse North	Sweden	SWE6	55.71	13.49	Ser	Ser
1719	Stensoffa, Kalvsmosse North	Sweden	SWE6	55.71	13.49	Ser	Ser
1721	Stensoffa, Ekskogen	Sweden	SWE6	55.69	13.46	Ser	Ser
1741	Torsby	Sweden	SWE2	60.19	12.74	Ser	Ser
1742	Torsby	Sweden	SWE2	60.19	12.74	Cys/Ser	Cys/Ser
1744	Torsby	Sweden	SWE2	60.17	12.76	Cys/Ser	Ser
1765	Strandkaer	Denmark	DNK1	56.23	10.57	Ser	Ser
1826	Calabria, Catena Costiera	Italy	IT3	39.30	16.12	Ser	Ser
1828	Calabria, Sila Grande	Italy	IT3	39.35	16.49	Ser	Cys/Ser
1845	Apulia	Italy	IT2	41.82	16.01	Ser	Ser
1848	Snasa	Norway	NOR2	64.17	12.05	Ser	Ser
1859	Venabygd	Norway	NOR5	61.58	10.06	Ser	Ser
1867	Eksingedalen	Norway	NOR4	60.79	5.97	Ser	Ser
1871	Eksingedalen	Norway	NOR4	60.79	5.97	Ser	Ser
1879	Lillesand	Norway	NOR6	58.24	8.23	Ser	Ser
1901	Goteborg	Sweden	SWE4	57.62	12.42	Ser	Ser
1903	Viterbo	Italy	IT1	42.52	12.12	Ser	Ser
1911	Armendarits	France	FRA7	43.30	1.17	Ser	Ser

Table S5. Allelic cytonuclear disequilibria for Europe (EU) and for the combination Europe and Britain (EU+GB). Results show the non-random association between β52Cys alleles at the nuclear locus and three major mtDNA lineages. Statistical significance (α = 0.05) was assessed using the asymptotic test. Normalised estimates take into account the bounds imposed by marginal frequencies of the two markers.

	HBB-T1 52Cys			HBB-T2 52Cys		
Disequilibria	Western	Eastern	Carpathian	Western	Eastern	Carpathian
EU estimate	0.084	-0.032	-0.037	0.075	-0.021	-0.039
Normalised estimate	0.381	-0.558	-0.256	0.667	-0.846	-0.615
EU + GB estimate	0.074	-0.031	-0.025	0.054	-0.014	-0.031
Normalised estimate	0.349	-0.624	-0.134	0.559	-0.830	-0.484

Table S6. Loadings of variables comprising principal components for the datasets containing data for continental Europe (EU), Britain (GB) and for the combined dataset (EU+GB). Loadings > |0.5| are highlighted.

	EU				GB		EU + GB			
Variable	PC1	PC2	PC3	PC4	PC1	PC2	PC1	PC2	PC3	PC4
AMT	0.470	-0.808	0.216	0.229	0.141	0.968	0.202	-0.874	0.367	0.187
TDrange	0.514	0.078	0.750	-0.210	0.931	-0.328	0.604	0.088	0.584	-0.377
Isotherm	0.107	-0.630	0.434	-0.365	0.431	-0.795	-0.180	-0.680	0.198	-0.445
Tseason	0.458	0.767	0.293	0.141	0.906	0.206	0.657	0.657	0.267	0.072
MaxTwarm	0.741	-0.335	0.514	0.224	0.839	0.487	0.709	-0.315	0.610	0.101
MinTcold	0.056	-0.964	-0.095	0.141	-0.550	0.790	-0.292	-0.925	0.016	0.144
TArange	0.521	0.651	0.488	0.041	0.956	-0.073	0.697	0.569	0.376	-0.055
MeanTwetQ	0.703	0.235	0.164	0.088	0.621	0.070	0.753	0.121	0.189	0.044
MeanTdryQ	-0.218	-0.768	-0.083	0.205	-0.729	0.490	-0.447	-0.671	0.059	0.241
MeanTwarmQ	0.719	-0.458	0.348	0.314	0.504	0.816	0.634	-0.466	0.531	0.249
MeanTcoldQ	0.140	-0.968	-0.006	0.125	-0.508	0.840	-0.220	-0.949	0.086	0.112
AP	-0.949	-0.049	0.259	0.141	-0.958	-0.139	-0.923	0.222	0.297	0.069
Pwet	-0.893	0.095	0.251	0.329	-0.977	-0.042	-0.837	0.349	0.315	0.243
Pdry	-0.842	-0.166	0.369	-0.246	-0.961	-0.199	-0.867	0.068	0.304	-0.305
Pseason	0.041	0.582	-0.074	0.666	-0.932	0.248	0.109	0.572	0.061	0.668
PwetQ	-0.900	0.088	0.245	0.328	-0.976	-0.059	-0.851	0.334	0.306	0.240
PdryQ	-0.868	-0.202	0.350	-0.177	-0.936	-0.251	-0.889	0.057	0.318	-0.250
PwarmQ	-0.695	0.382	0.387	-0.062	-0.844	-0.400	-0.538	0.622	0.341	-0.174
PcoldQ	-0.900	-0.217	0.115	0.211	-0.983	0.016	-0.933	0.016	0.182	0.181
% explainedo	41.4	29.0	11.4	6.9	65.3	24.0	42.9	29.1	10.8	7.0
cumulative %		70.5	81.9	88.7		89.3		72.0	82.8	89.8

o percentage of total variance explained by the component

Table S7. Spearman's rho and associated *p*-value for correlation between HBB-T1 52Cys allele frequency and latitude, longitude, altitude and principal components identified in PCA. Results shown for the datasets of continental Europe (EU), Britain (GB) and for the combined dataset (EU+GB). $p < 0.05$ is underlined. For variables comprising the principal components and their loadings see Table 6.

	EU		GB		EU + GB	
Component	rho	*p*	rho	*p*	rho	*p*
Latitude	-0.1042	0.3907	-0.8810	0.0002	-0.1374	0.2182
Longitude	-0.3298	0.0053	0.2792	0.3795	-0.3324	0.0023
Altitude	-0.2449	0.0410	0.1414	0.6611	-0.2637	0.0167
PC1	0.2074	0.0850	0.3444	0.2729	0.0754	0.5009
PC2	-0.3996	0.0006	0.8194	0.0011	-0.5050	0.000001
PC3	-0.0040	0.9735			-0.0528	0.6377
PC4	-0.3661	0.0018			-0.2485	0.0243

Table S8. Result from Samβada analysis for continental Europe dataset, 1 variable models. Only significant models with significant parents are shown (according to Wald score). Bonferroni adjusted *p*-value associated with Wald score is $p = 0.00044$.

Table S9. Result from Samβada analysis for continental Europe dataset, 2 variable models. Only significant models with significant parents are shown (according to Wald score). Bonferroni adjusted *p*-value associated with Wald score is $p = 0.000039$.

Table S10. Result from Samβada analysis for continental Europe dataset, 3 variable models. Only significant models with significant parents are shown (according to Wald score). Bonferroni adjusted *p*-value associated with Wald score is $p = 0.0000057$.

Table S11. Result from Samβada analysis for continental Europe dataset, 4 variable models. Only significant models with significant parents are shown (according to Wald score). Bonferroni adjusted *p*-value associated with Wald score is $p = 0.0000011$.

Table S12. Result from Samβada analysis for Britain dataset, 1 variable models. Only significant models with significant parents are shown (according to Wald score). Bonferroni adjusted *p*-value associated with Wald score is $p = 0.00044$.

Table S13. Result from Samβada analysis for Britain dataset, 2 variable models. Only significant models with significant parents are shown (according to Wald score). Bonferroni adjusted *p*-value associated with Wald score is $p = 0.000039$.

Table S14. Result from Samβada analysis for Britain dataset, 3 variable models. Only significant models with significant parents are shown (according to Wald score). Bonferroni adjusted *p*-value associated with Wald score is $p = 0.0000057$.

Table S15. Result from Samβada analysis for combined dataset (Britain + continental Europe), 1 variable models. Only significant models with significant parents are shown (according to Wald score). Bonferroni adjusted *p*-value associated with Wald score is $p = 0.00044$.

Table S16. Result from Samβada analysis for combined dataset (Britain + continental Europe), 2 variable models. Only significant models with significant parents are shown (according to Wald score). Bonferroni adjusted *p*-value associated with Wald score is $p = 0.000039$.

Table S17. Result from Samβada analysis for combined dataset (Britain + continental Europe), 3 variable models. Only significant models with significant parents are shown (according to Wald score). Bonferroni adjusted *p*-value associated with Wald score is $p = 0.0000057$.

Table S18. Result from Samβada analysis for combined dataset (Britain + continental Europe), 4 variable models. Only significant models with significant parents are shown (according to Wald score). Bonferroni adjusted *p*-value associated with Wald score is $p = 0.0000011$.

Table S19. Result from Samβada analysis for Western and Carpathian clades (as defined by mtDNA phylogeography), 1, 2 and 3 variables models. Only significant models with significant parents are shown (according to Wald score). Bonferroni adjusted *p*-value associated with Wald score is $p = 0.00044$, $p = 0.000039$, $p = 0.0000057$ respectively.

Due to large size, the tables are provided in separate Excel file Tables S8-S19_Supporting_information.xlsx, each table on separate sheet.

Table S20. Alignment of HBB-T1 and HBB-T2 haplotypes, showing variable sites only. Identified gene conversion tracts are shown. HBB-T1 haplotypes are in grey, HBB-T2 haplotypes in white. Coding regions of the beta globin gene are marked by black boxes. Ser52Cys position is marked by orange column, position determining complete/incomplete Chi sequence is marked by red box. Conversion tracts identified by method of Betrán *et al.* (2007) are in grey/white depending on their gene of origin. Conversion tracts identified by GENECONV are in yellow.

Due to the large size of this table, it is provided as a separate Excel file, Table S20_Supporting_information.xlsx.

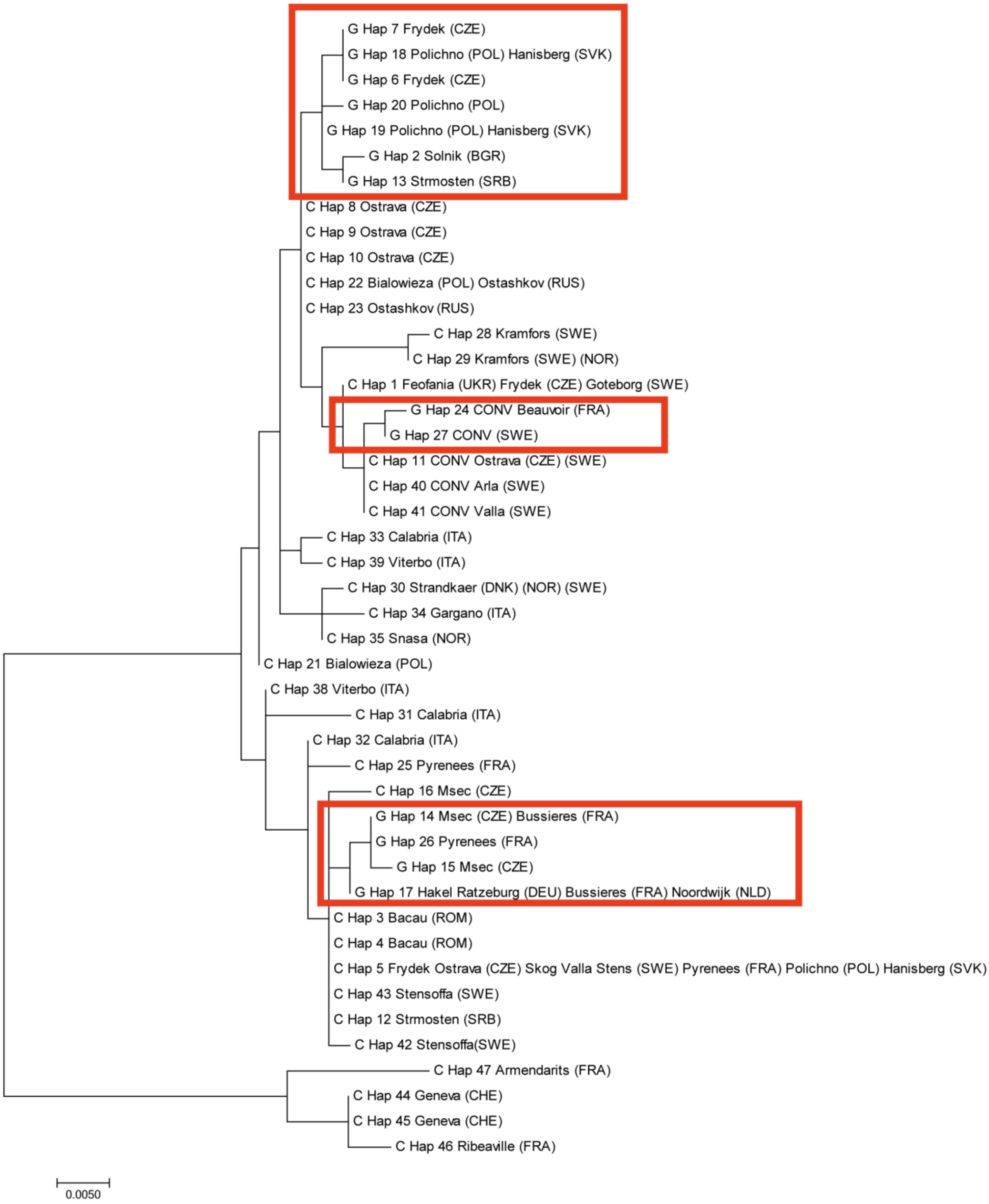

Figure S1. Maximum likelihood phylogeny of HBB-T1 haplotypes based on the alignment segment left of the breakpoint at site 521. C and G letters represent the Ser and Cys allele, respectively. Haplotypes containing the Cys allele are boxed.

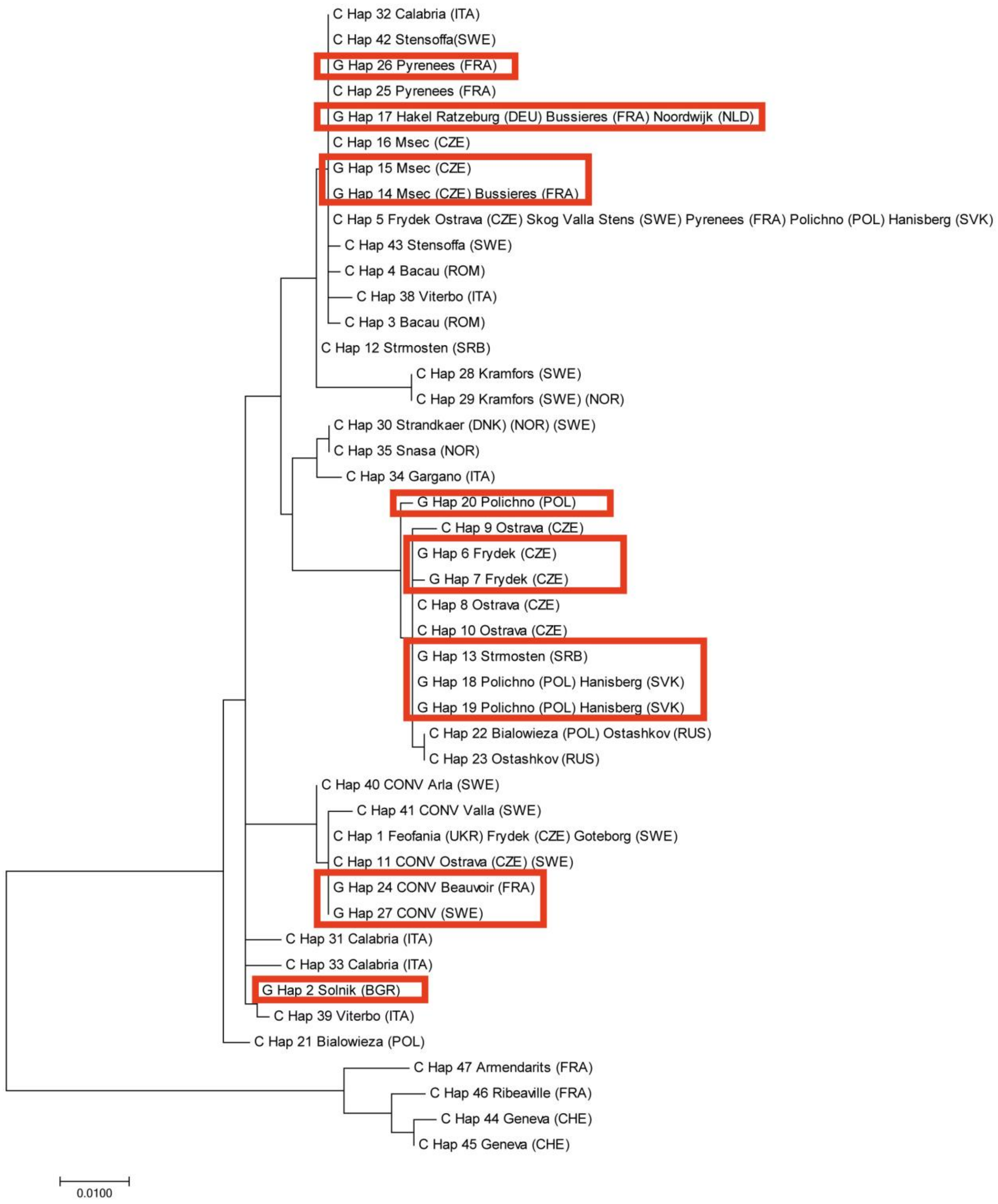

Figure S2. Maximum likelihood phylogeny of HBB-T1 haplotypes based on the alignment segment right of the breakpoint at site 521. C and G letters represent the Ser and Cys allele, respectively. Haplotypes containing the Cys allele are boxed.

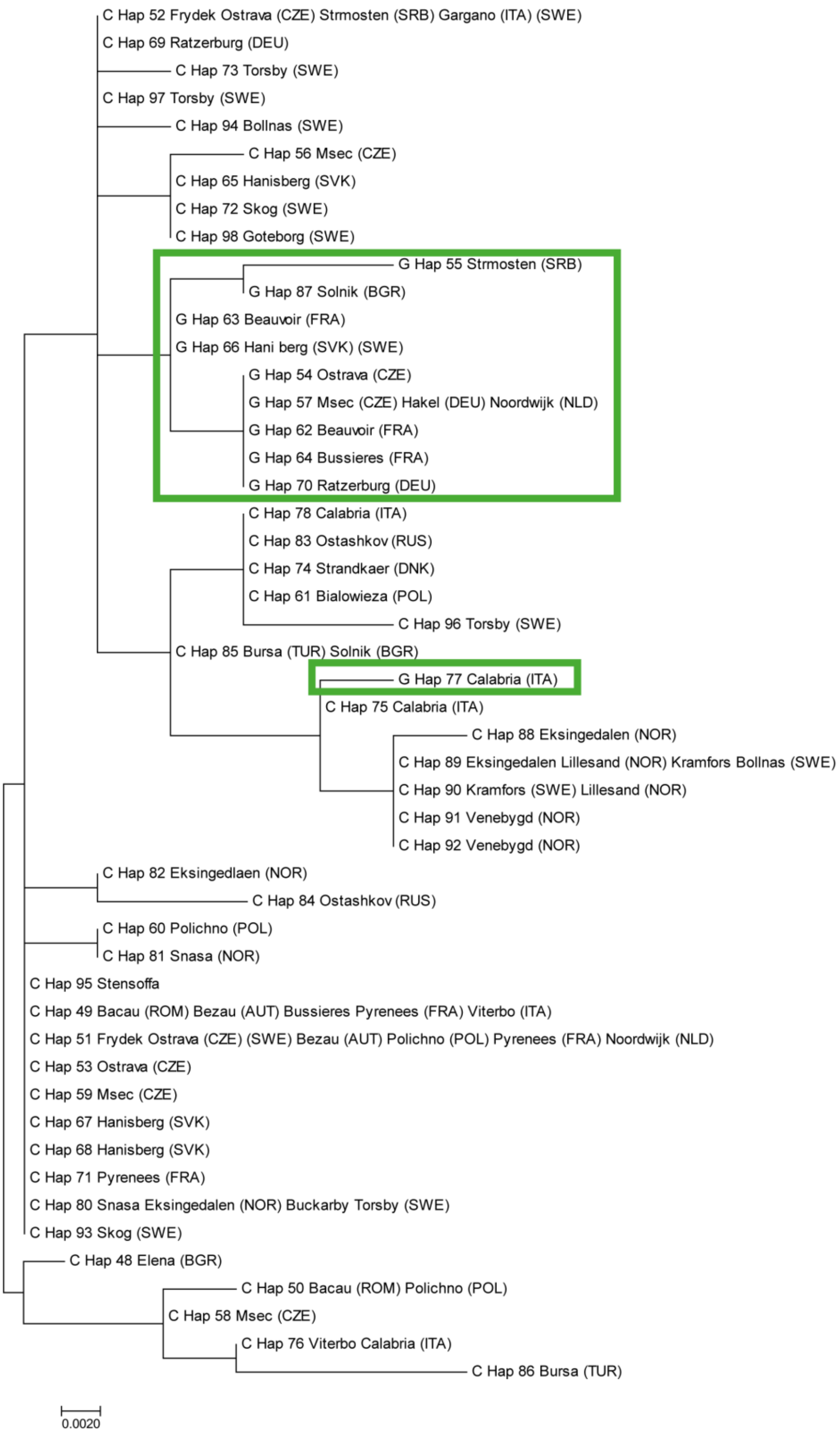

Figure S3. Maximum likelihood phylogeny of HBB-T2 haplotypes based on the alignment segment left of the breakpoint at site 329. C and G letters represent the Ser and Cys allele, respectively. Haplotypes containing the Cys allele are boxed.

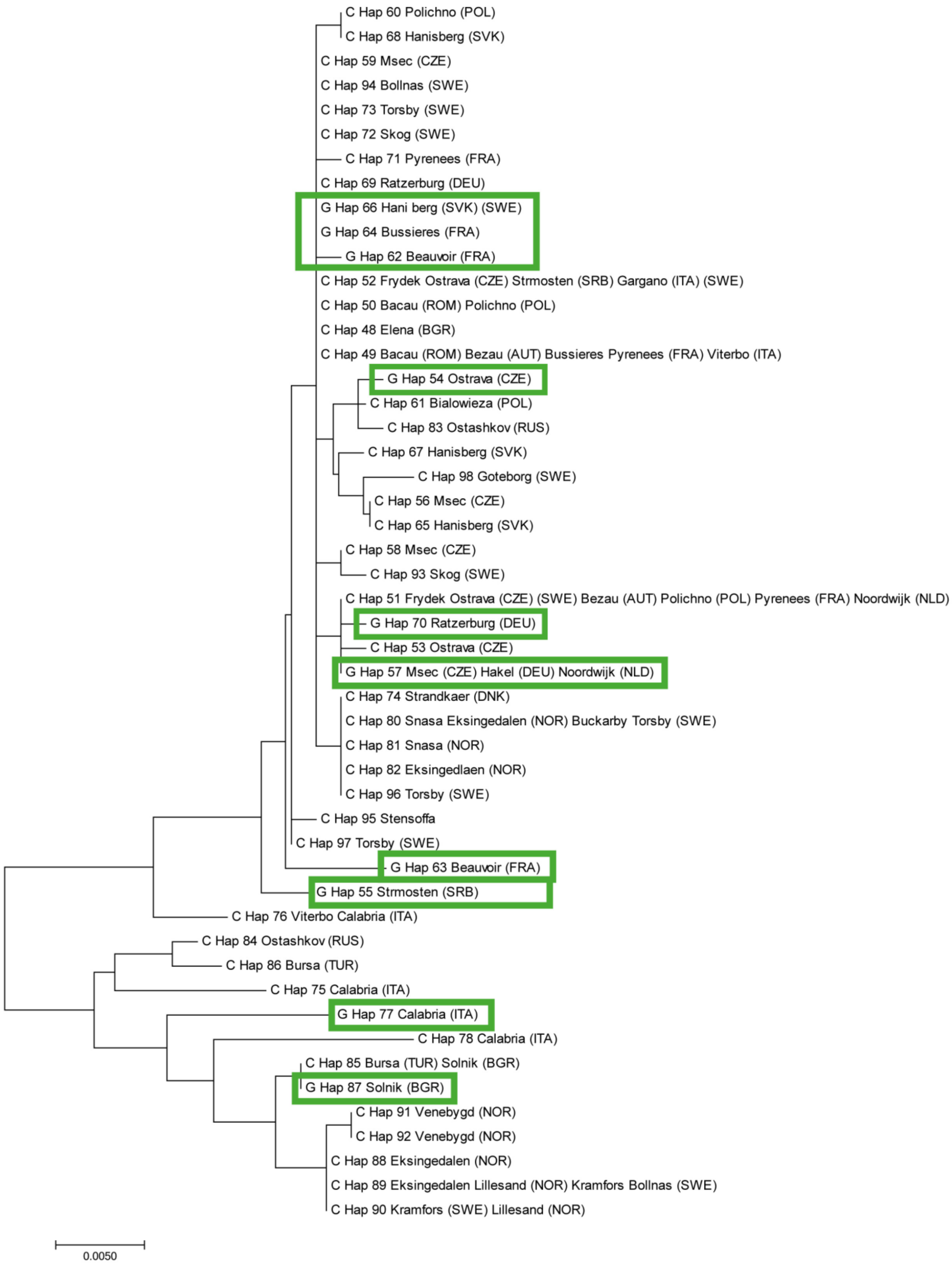

Figure S4. Maximum likelihood phylogeny of HBB-T2 haplotypes based on the alignment segment right of the breakpoint at site 329. C and G letters represent the Ser and Cys allele, respectively. Haplotypes containing the Cys allele are boxed.

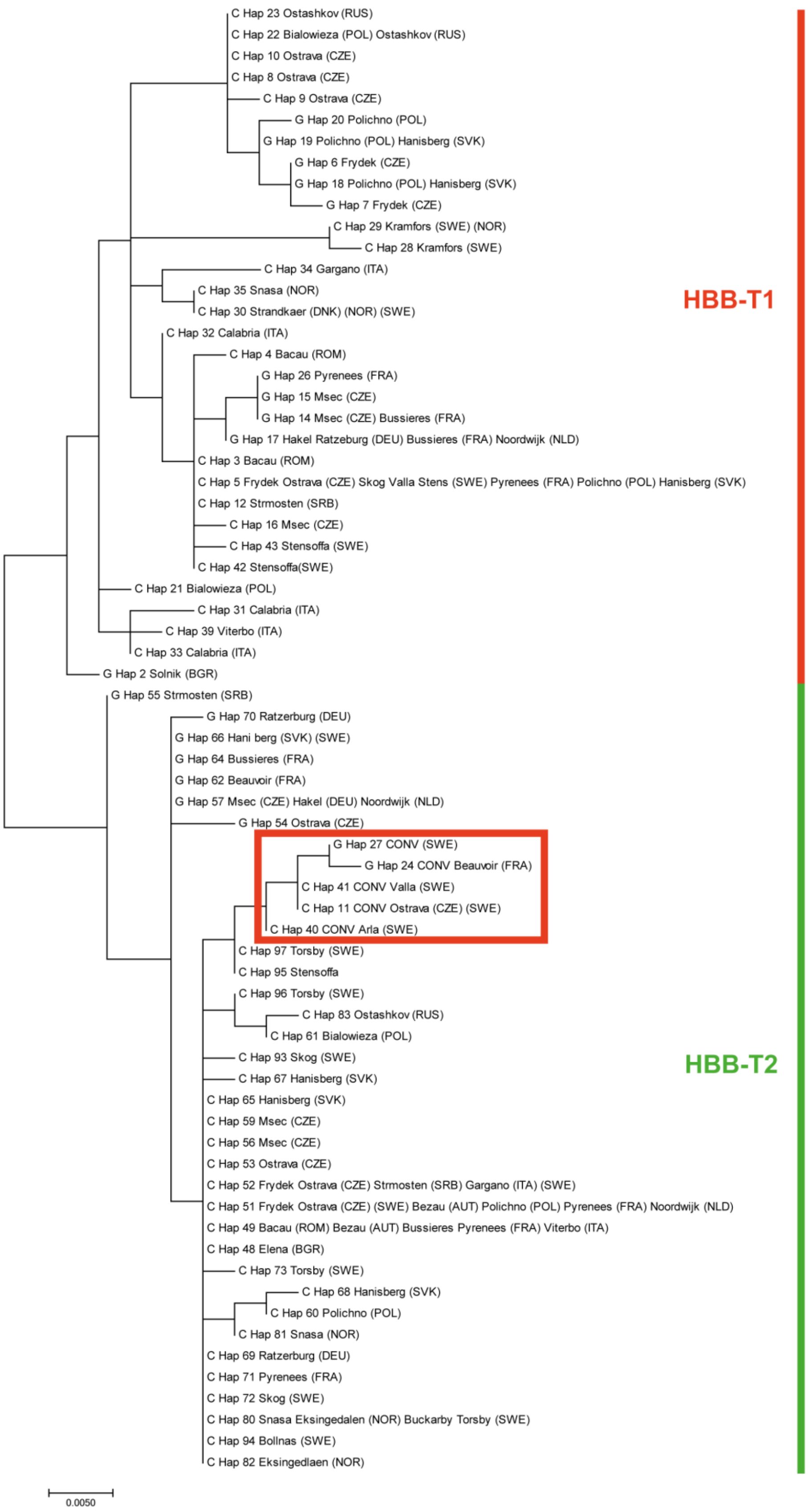

Figure S5. Maximum likelihood phylogeny for both genes representing the converted gene segment spanning sites 208 - 664. Other haplotypes containing conversion tracts are excluded. C and G letters represent the Ser and Cys allele, respectively. Converted haplotypes are boxed.

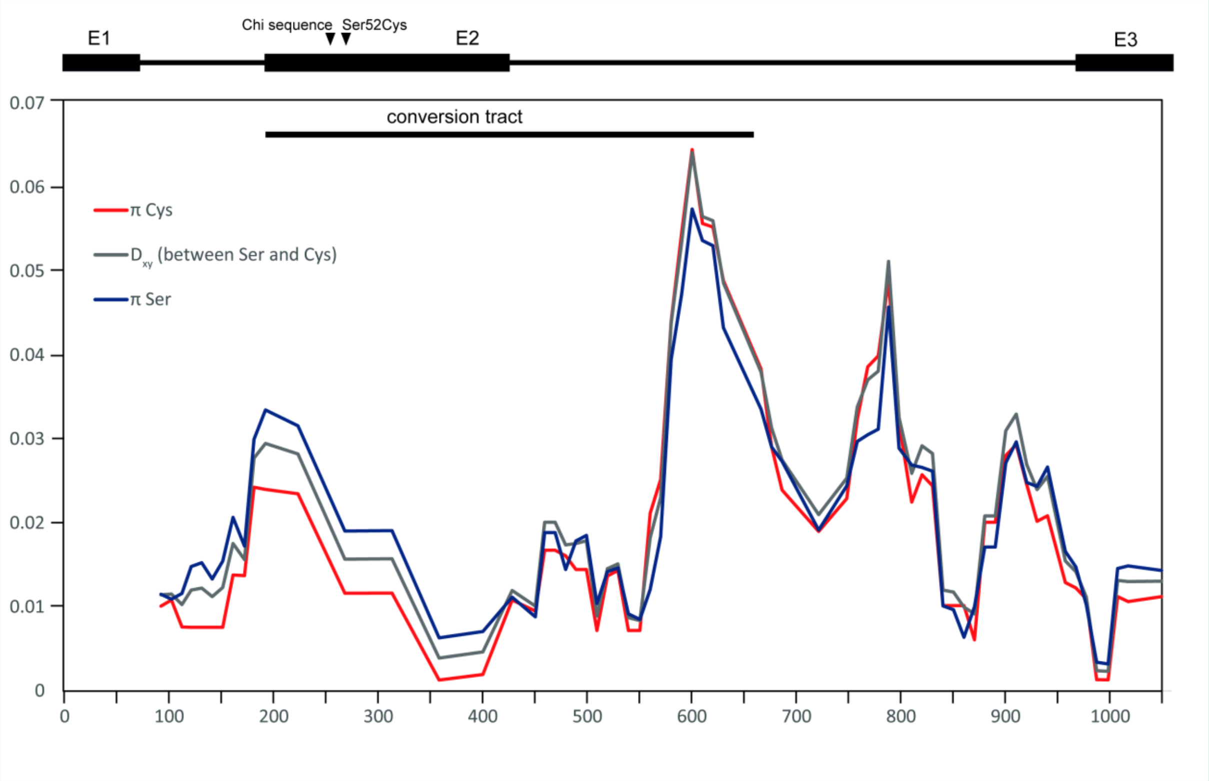

Figure S6. Results of a sliding window test of silent site diversity within (π) and between (D_{xy}) the Cys and Ser haplotypes. Exons (E1-3) are marked by black rectangles (top).
